# Diversification and coevolution of the ghrelin/growth hormone secretagogue receptor system in vertebrates

**DOI:** 10.1002/ece3.2057

**Published:** 2016-03-14

**Authors:** Mbaye Tine, Heiner Kuhl, Peter R. Teske, Matthias H. Tschöp, Martin Jastroch

**Affiliations:** ^1^ Genome Centre at Max Planck Institute for Plant Breeding ResearchCarl‐von‐Linné‐Weg 10D‐50829KölnGermany; ^2^ Molecular Zoology LaboratoryDepartment of ZoologyUniversity of JohannesburgKingsway CampusAuckland Park 2006South Africa; ^3^ Max Planck Institute for Molecular GeneticsIhnestrasse 63‐7314195BerlinGermany; ^4^ Helmholtz Diabetes Center & German Diabetes Center (DZD)Helmholtz Zentrum München, 85764 Neuherberg, Germany; Division of Metabolic DiseasesTechnische Universität München80333MunichGermany

**Keywords:** Coevolution, divergence, function, ghrelin, growth hormone secretagogue receptor, vertebrates

## Abstract

The gut hormone ghrelin is involved in numerous metabolic functions, such as the stimulation of growth hormone secretion, gastric motility, and food intake. Ghrelin is modified by ghrelin O‐acyltransferase (*GOAT)* or membrane‐bound O‐acyltransferase domain‐containing 4 (*MBOAT4*) enabling action through the growth hormone secretagogue receptors (*GHS‐R*). During the course of evolution, initially strong ligand/receptor specificities can be disrupted by genomic changes, potentially modifying physiological roles of the ligand/receptor system. Here, we investigated the coevolution of ghrelin, *GOAT,* and *GHS‐R* in vertebrates. We combined similarity search, conserved synteny analyses, phylogenetic reconstructions, and protein structure comparisons to reconstruct the evolutionary history of the ghrelin system. Ghrelin remained a single‐gene locus in all vertebrate species, and accordingly, a single *GHS‐R* isoform was identified in all tetrapods. Similar patterns of the nonsynonymous (*dN*) and synonymous (*dS*) ratio (*dN/dS*) in the vertebrate lineage strongly suggest coevolution of the ghrelin and *GHS‐R* genes, supporting specific functional interactions and common physiological pathways. The selection profiles do not allow confirmation as to whether ghrelin binds specifically to *GOAT*, but the ghrelin *dN/dS* patterns are more similar to those of *GOAT* compared to *MBOAT1* and *MBOAT2* isoforms. Four *GHS‐R* isoforms were identified in teleost genomes. This diversification of *GHS‐R* resulted from successive rounds of duplications, some of which remained specific to the teleost lineage. Coevolution signals are lost in teleosts, presumably due to the diversification of *GHS‐R* but not the ghrelin gene. The identification of the *GHS‐R* diversity in teleosts provides a molecular basis for comparative studies on ghrelin's physiological roles and regulation, while the comparative sequence and structure analyses will assist translational medicine to determine structure–function relationships of the ghrelin/*GHS‐R* system.

## Introduction

Complex physiological networks are integrated through ligand/binding‐receptor physical interactions (Howard et al. 1996; Kaiya et al. 2014a) to regulate essential physiological functions such as growth, reproduction, behavior, homeostasis, and metabolism (Tschöp et al. 2000; Asakawa et al. 2005; Chen et al. 2009; Kojima and Kangawa 2010; Kitazawa et al. 2012; Müller et al. 2015). G protein‐coupled receptors, the largest membrane receptors of all living organisms (Vaudry 2014), form a subset of such a system (Kaiya et al. 2013a, 2014a). The activation of heterotrimeric G protein mediates the actions of signaling proteins such as neuroendocrine peptides by allowing the translation of extracellular signals into the intracellular space (Kaiya et al. 2008, 2014a). Receptors and their peptide ligands originated from common ancestral genes and may have diversified through gene duplication (Meyer and Schartl 1999; Meijer et al. 2007) (Miki et al. 1992; Chan and Cheng 2004), retrotransposition (Maxwell 2014), or alternative splicing to eventually become a gene family. Whole‐genome duplication (*WGD*) has been considered as the most common mechanism resulting in the diversification of ligand and receptor gene families (Taylor et al. 2003). Especially in teleosts, the whole genome has undergone a teleost‐specific *WGD* (Meyer and Schartl 1999; Meyer and Van de Peer 2005). Many ligand and binding‐receptor duplicates have been preserved in the genome after duplication through subfunctionalization or neo‐functionalization either maintaining the original function or evolving new functions (Zhang and Cohn 2008). Theories on gene duplication stipulate that new duplicates are usually redundant and one of the two paralogous duplicates is free of selective constraints, accumulating deleterious mutations to become nonfunctional pseudogene (Kellogg 2003; Zhang and Gaut 2003) and eventually being deleted from the genome (Wagner 1998; Zhang and Gaut 2003). In rare cases, both paralogous duplicates are maintained active because they differ in their functional aspects (Nowak et al. 1997; Zhang and Gaut 2003; Hughes 2005). After the duplication event, endogenous ligand duplicates have to maintain their properties to selectively recognize and bind to their specific receptors, and any alteration in their sequences may compromise this recognition (Tillier and Charlebois 2009b). This implies that the evolution of the protein ligand structure should be correlated with the evolution of their binding proteins, which can be studied as ligand–receptor coevolution (Kaiya et al., 2013c). The coevolution of orthologs and/or paralogs can be investigated using phylogenetic reconstruction and evolutionary distance approaches (Tillier and Charlebois 2009b; Chan et al. 2013). The phylogenetic approach is based on the fact that orthologs with similar phyletic patterns may have coevolved during the course of evolution, while the group of orthologs with different phylogenetic profiles may have evolved differently (Koszul et al. 2004; Chen et al. 2009; Cohen et al. 2012). The evolutionary distance measured by average *dN/dS* differences between ortholog pairs is used to identify genes that may have coevolved (Tillier and Charlebois 2009a). This approach relies on the principle that the evolutionary rate of protein coding genes is strongly related to their functions (Chan et al. 2013). While there are initially strong ligand/receptor specificities, such interactions can be disrupted during the course of evolution through evolutionary changes including duplication, divergence, rearrangement, and recombination (Laisney et al. 2010). The interaction specificities can persist if both ligands and receptors have undergone similar evolutionary constraints and if they have coevolved after these evolutionary events have occurred (Moyle et al. 1994; van Kesteren et al. 1996). Many structural features of proteins such as the position of the transmembrane domain (*TMD*), *α*‐helix or *β*‐strand in the secondary or tertiary structure, and relative solvent accessibility (*RSA*) are known to influence their evolution (Bustamante et al. 2000; Ramsey et al. 2011). The *RSA,* which measures the extend amino acid residue exposure, has been particularly used to investigate the biophysical and evolutionary properties of proteins (Shaytan et al. 2009; Tien et al. 2013). It is well established that the *RSA* correlates with the evolutionary rates of proteins (Goldman et al. 1998; Bloom et al. 2006; Franzosa and Xia 2009; Tien et al. 2013), especially for G protein‐coupled receptors, which mediate the actions of various protein ligands (Spielman and Wilke 2013).

Ghrelin is a gastrointestinal peptide belonging to the same family as the motilin genes. Secreted from the stomach, ghrelin is involved in various physiological processes including appetite regulation, food uptake, and glucose metabolism (Müller et al. 2015). Besides neuroendocrine and cardiovascular functions, ghrelin action has also been implicated in cell differentiation and proliferation (Asakawa et al. 2005; Chen et al. 2009; Kaiya et al. 2013b). The mature ghrelin peptide is modified specifically at the animo acid residue serine‐3 by the ghrelin O‐acyltransferase (*GOAT*) enzyme (Kitazawa et al. 2015). Ghrelin acylation is required to accomplish all of its physiological activities. The actions of ghrelin are mediated by growth hormone secretagogue receptor (*GHS‐R*), a G‐coupling protein receptor with seven *TMDs* (Howard et al. 1996; Kaiya et al. 2013c). Ghrelin binds to the *TMD*, and ghrelin/*GHS‐R* signaling increases intracellular Ca^2+^ concentration (Howard et al. 1996).

While only one ghrelin isoform has been so far described in all vertebrates (Kaiya et al. 2011b), several *GHS‐R* variants were identified in many species (Kaiya et al. 2003, 2010, 2014a, 2014b). Two *GHS‐R* isoforms were characterized in mammals based on their amino acid sequence composition and length. *GHS‐Ra* is derived from regular splicing and is functionally active, whereas *GHS‐Rb*, whose function is not well understood, results from alternative splicing (Howard et al. 1996). Nonmammalian vertebrates other than fishes possess *GHS‐Ra* which is similar to the *GHS‐Ra* of mammals and is activated by the ghrelin gene (Kaiya et al. 2011a, 2013a, 2013b). Previous studies support that the *GHS‐Ra* was duplicated in some fish species and is subdivided into two paralogs, *GHS‐R1a* and *GHS‐R2a*. While *GHS‐R1a* is more similar to mammalian *GHS‐Ra*,* GHS‐R2a* is more different but nevertheless possesses the functional domain which is activated by the ghrelin peptide (Kaiya et al. 2013a, 2014a). Another isoform named *GHS‐R1a‐like* (*GHS‐R1a‐LR*) has been so far found only in fishes (Kaiya et al. 2013a, 2014a).

The ghrelin/*GHS‐R* system offers an excellent opportunity to investigate the coevolution of ligands and their binding receptors. Why do teleosts only have a single ghrelin isoform, but three or more *GHS‐R* isoforms? Are all these *GHS‐R* isoforms functional and activated by the same ghrelin isoform? If the diversification of *GHS‐R* has resulted from teleost‐specific *WGD*, was the ghrelin gene affected by the same evolutionary events? Were certain ghrelin duplicates subsequently lost after duplications and did this happen due to functional redundancy? Were *GHS‐R* duplicates maintained by evolving new functions? All these questions can only be addressed by first investigating whether the ghrelin gene and its receptors have coevolved in all vertebrates. The functional triangle consisting ghrelin, its activating enzyme (*GOAT*) and the binding receptors (*GHS‐R*) support the hypothesis that these genes may have coevolved. The main objective of this study aimed to gain insights into the evolutionary dynamics of the ghrelin/*GHS‐R* system and to determine whether the ligand ghrelin coevolved with its binding receptors and activating enzyme *GOAT* during the course of vertebrate evolution. To this end, we first searched and identified all orthologous and paralogous isoforms of ghrelin, *GOAT* (also known as *MBOAT4*) and the *GHS‐R* in a broad range of species covering all vertebrate lineages. We compared the selection patterns of ghrelin/*GHS‐R* and ghrelin/*GOAT,* as well as the selection patterns between ghrelin and other *MBOAT* isoforms (*MBOAT1/2)* to determine whether these patterns are different from the *MBOAT4* selection profiles. We then performed whole‐genome comparative analyses including similarity search, conserved synteny analyses, phylogenetic reconstructions, and protein secondary structure comparisons. These analyses predict scenarios of coevolution and the interaction within the ghrelin/*GHS‐R* system. Our analysis uncovers the functional diversification of *GHS‐R* and may assist in the understanding of the physiological roles in diverse vertebrate lineages, in particular the teleost fishes.

## Materials and Methods

### Identification of ghrelin ligand and *GHS‐R*

Protein sequences of *GHS‐R* of the zebrafish, *Danio rerio,* and of humans, *Homo sapiens,* were blasted against the NCBI nonredundant database to identify orthologs and paralogs in vertebrate species. The sequence information from NCBI was complemented by BLAST similarity search in species whose complete genome is available in the ENSEMBL Genome Browser. Two *GHS‐R* loci were considered as paralogs or orthologs when the two corresponding protein sequences matched on aligned blocks with an average length of at least 80% with ≥70% identity. Synteny‐based analyses were then performed to confirm whether the genes identified are real *GHS‐R* paralogs or orthologs. These synteny analyses consisted of performing a comprehensive comparative analysis of the genomic region harboring *GHS‐R* genes to identify their upstream and downstream flanking genes. When a *GHS‐R* gene was not identified in a given species by similarity search using the protein sequence of its ortholog, the sequences of flanking genes were used for its identification by means of BLAST search. When the flanking genes were identified and no *GHS‐R* was predicted between them, the genomic region potentially harboring these flanking genes was extracted and re‐annotated using de novo and/or similarity‐based annotation approaches. A gene annotated by de novo or similarity‐based approach was considered to be a *GHS‐R* locus when it matched the well‐characterized *GHS‐R* protein sequences on aligned blocks with an average length of at least 80% with ≥70% identity. The protein sequences of predicted genes from the de novo annotation were then confirmed as *GHS‐R* by BLAST against the well‐characterized *GHS‐R* genes using the above criteria. The same similarity and synteny search criteria (aligned blocks of protein sequences that match with an average length of at least 80% with ≥70% identity, re‐annotation of genomic regions potentially harboring orthologs) were applied to identify ghrelin orthologs in NCBI databases and in all vertebrate species whose genome is present in the ENSEMBL Genome Browser. Likewise, the same similarity search approach was used to identify *MBOAT4* (*GOAT*) and *MBOAT1/2* orthologs and paralogs in a wide range of vertebrate species. The sequences of *MBOAT* isoforms were used in this study only for the evolutionary investigation, that is, the comparison of *dN/dS* patterns.

### Phylogenetic analyses

The ghrelin and *GHS‐R* phylogenetic trees of both mammalian and nonmammalian vertebrates were reconstructed using protein sequences of species belonging to these respective lineages. The protein sequences of *GHS‐R* isoforms from a broad range of mammalian species were aligned using MAFFT version 7 (Katoh and Standley 2013). The protein sequences of *GHS‐R* of nonmammalian vertebrates, and the ghrelin protein sequences of all vertebrates analyzed in this study, were also separately aligned using MAFFT. The Gblocks Server was used to improve these alignments (Castresana 2000; Talavera and Castresana 2007). The well‐aligned blocks identified by Gblock software were then used to reconstruct a phylogenetic tree using MEGA software version 6 (Tamura et al. 2013). The maximum‐likelihood method with the Jones–Taylor–Thornton (*JTT*) substitution model was used to constructed the phylogenetic trees, which was rooted with the reptile *GHS‐R* protein sequence (*Chelonia myda*s and *Pelodiscus sinensis*) for mammal tree and lamprey (*Petromyzon marinus*) *GHS‐R* protein sequence for nonmammal tree. The vertebrate ghrelin tree was rooted using the midpoint rooting approach. The *GHS‐R* actually belongs to the same family as motilin (*MLN‐R*). These two receptors share some homologies and might be mixed up when considering only similarity search by BLAST. We therefore conducted phylogenetic reconstruction of a tree including *MLN‐R* genes from teleost and tetrapods.

### Evolutionary analyses

The *dN/dS* by site was used to measure the selective pressure exerted on ghrelin, *GOAT*,* MBOAT1/2,* and *GHS‐R* genes. The *dN/dS* ratio is a common method used to measure the evolutionary selective pressure exerted on genes. It is commonly accepted that the theoretical limit between positive and negative selection is a *dN/dS* ratio of one. A *dN/dS* ratio less than one is indicative of negative selection whereas a ratio greater than one is a sign of positive selection. Pairwise comparisons of *dN/dS* ratios were conducted between vertebrate ghrelin orthologs using nucleotide sequences, and also between vertebrate *GHS‐R* orthologs and paralogs. Similar pairwise comparisons of the average *dN/dS* of *GOAT* and *MBOAT1/2* orthologs were also conducted. The *dN/dS* ratios between *GHS‐R* paralogs and orthologs were calculated using both naive empirical Bayes and Bayes empirical Bayes model implemented in JCoDA (Steinway et al. 2010). There are several methods incorporated in the JCoDA software (Department of Biology, The College of New Jersey, Ewing, USA) for the estimation of *dN/dS* ratios, which include *NG* (Nei and Gojobori 1986), *YN* (Yang and Nielsen 2008a), and *LPB* (Li 1993; Pamilo and Bianchi 1993). All these methods were applied, and the results did not differ significantly. Finally, the *NG* method was applied and a Fisher's exact test was used to test the significance of differences in *dN/dS < *1 and *dN/dS > *1. The multiple‐comparisons Turkey's test was used to assess the significance of differences in the average *dN/dS* ratios of ghrelin orthologs between lineages. The same test was used to evaluate the significance of the average *dN/dS* ratios between *GHS‐R* and *MBOAT* clusters and between lineages. Sliding window *dN/dS* was used to identify codons of the *GHS‐R* and ghrelin proteins that are under positive selection. Codons that are under selective constraints were graphically visualized using the graph sliding *dN/dS* window option as implemented in JCoDA software. The size of the window was set at 200 bp, with a jump of 25 bp between windows.

### Protein structure prediction

The SABLE server (Adamczak et al. 2004) was used for the protein structure prediction, which included finding the number of *TMD*s, predicting the secondary structure, quantifying the *RSA* of amino acid residues along the protein sequences, and identifying physical–chemical property profiles. The *RSA* measures the solvent surface accessible to amino acid residues in a protein. The *RSA* represents the solvent‐accessible surface areas normalized by the surface area of the residue in the unfolded state. A *RSA* value of 0 means completely buried whereas a value of 9 is indicative of a fully exposed surface area. The predicted structures were visualized using the POLYVIEW‐2D viewer (Porollo et al. 2004). Correlations between *RSA* and other structural features of the protein, including *α*‐helix, *β*‐strand, and coil structures, were investigated; as were correlations between *RSA* and *dN/dS* ratios.

## Results

### Mammalian ghrelin and its orthologs in other vertebrates

**Figure 1:**
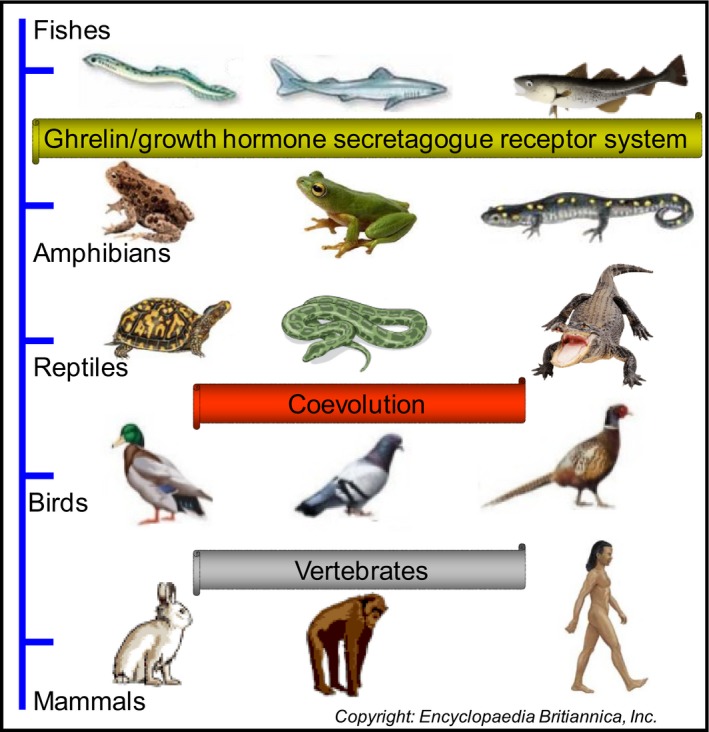
Graphical representation of the main vertebrate lineages studied here with regard to the coevolution of ghrelin/ghrelin receptor system.

**Figure 2:**
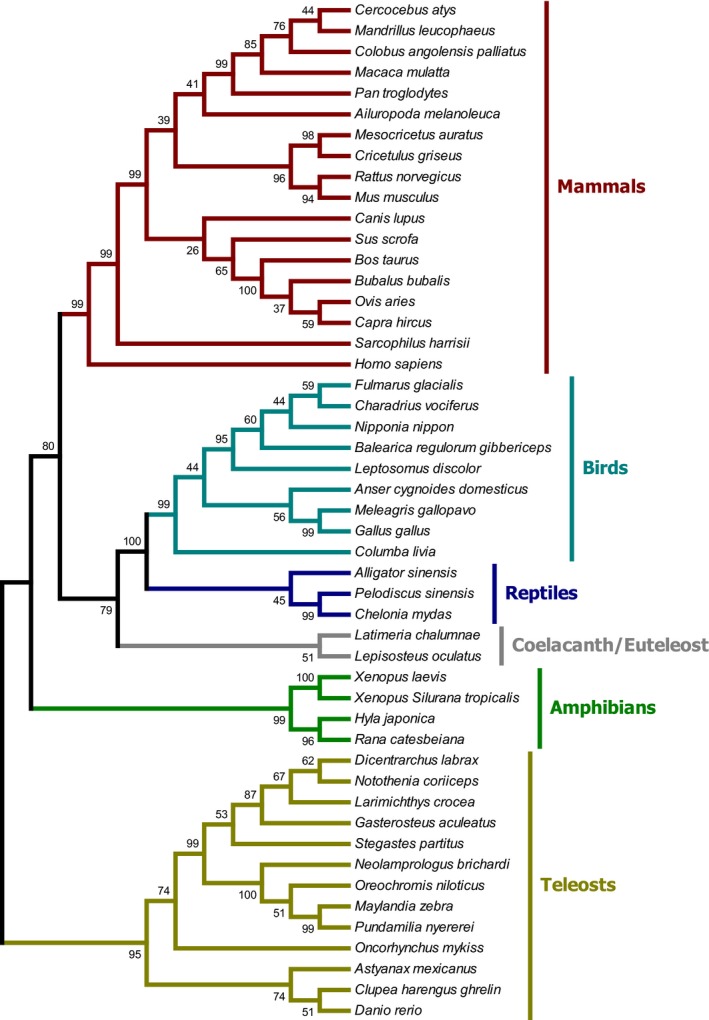
ML (with Jones–Taylor–Thornton (*JTT*) substitution model) phylogenetic tree of ghrelin gene in vertebrates. The tree was constructed with protein sequences and rooted using the midpoint rooting approach.

**Figure 3:**
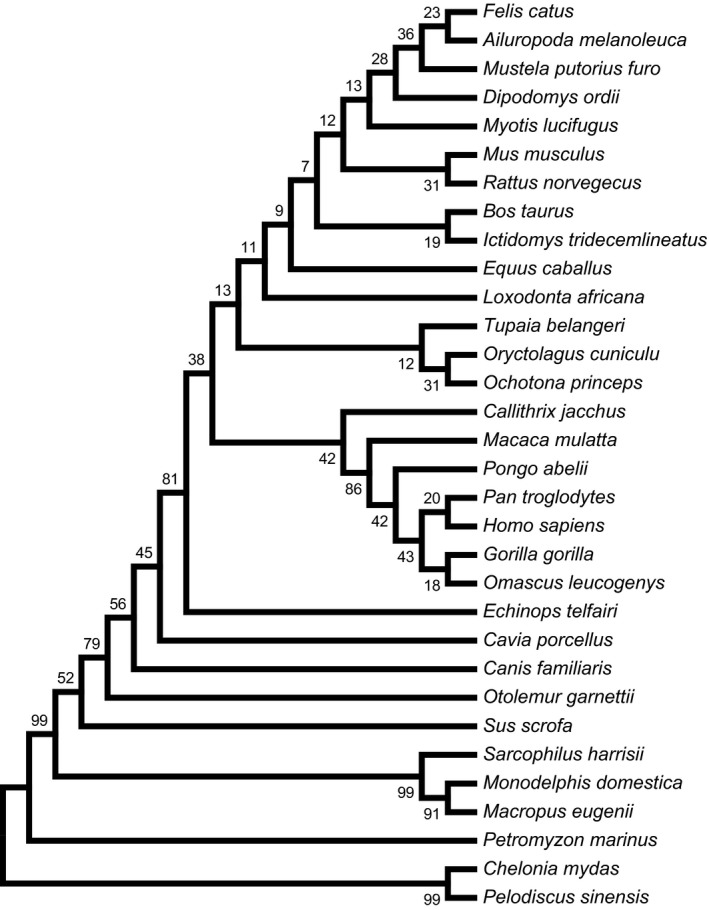
ML phylogenetic tree of *GHS‐R* in mammal vertebrates. The tree was constructed with protein sequences using the Jones–Taylor–Thornton (*JTT*) substitution model. The tree was rooted with the reptile *GHS‐R* protein sequence (*Chelonia myda*s and *Pelodiscus sinensis*).

**Figure 4:**
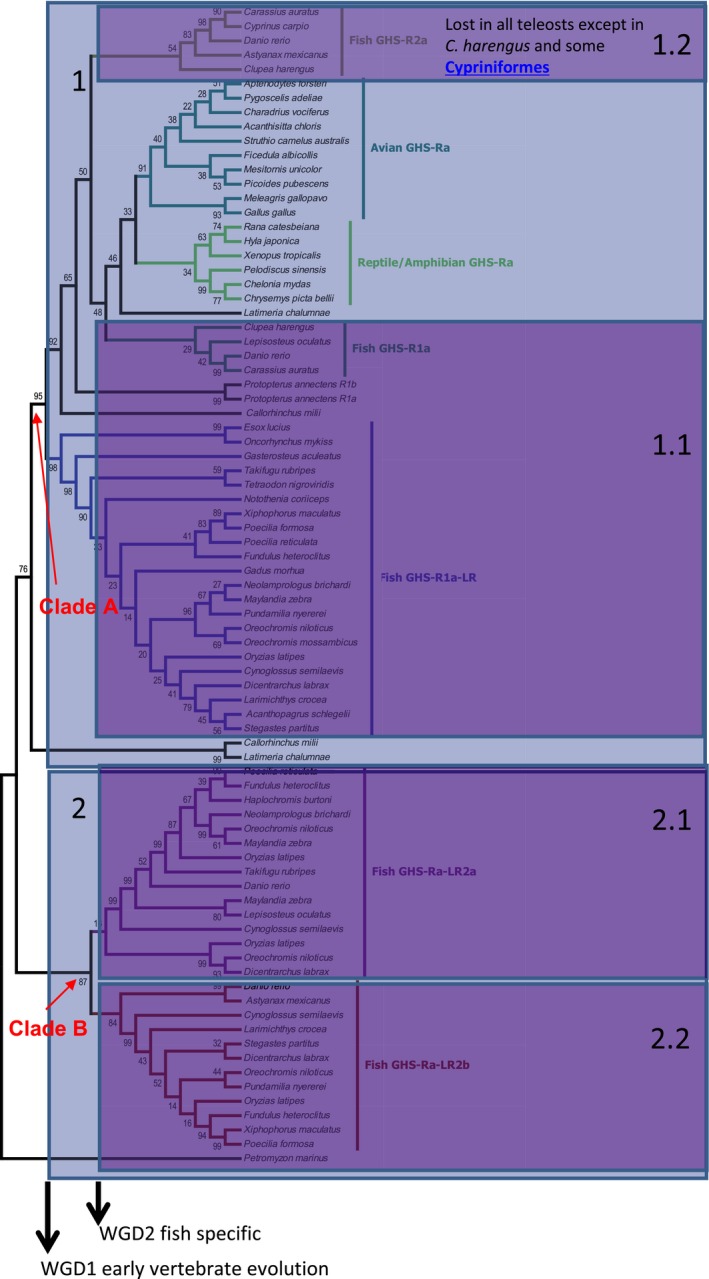
ML phylogenetic tree of *GHS‐R* in nonmammal vertebrates constructed with protein sequences and using the Jones–Taylor–Thornton (*JTT*) substitution model. The tree was rooted with the lamprey (*Petromyzon marinus*) *GHS‐R* protein sequence. WGD1: whole‐genome duplication 1; WGD2: whole‐genome duplication 2.

There are currently about 100 mammalian, 50 avian, and 50 fish genomes deposited at NCBI. This study that aimed on investigating the coevolution of the ghrelin/GHS‐R system in the main vertebrate lineages. The species selected for represent a fraction for which ghrelin/GHSR annotations were found in NCBI protein database using similarity search (Fig. 1). The selection was also based on the availability of completely sequenced genomes that allowed performing conserved synteny analyses in ENSEMBL. The similarity search revealed a single ghrelin gene encoding for a single isoform in mammals and nonmammalian vertebrates such as birds, reptiles, amphibians, teleosts, euteleosts, and the coelacanth. The selection of species can be retrieved from the phylogenetic tree (Figs. 2, 3, 4). The conserved synteny analyses revealed that the upstream and downstream flanking genes of ghrelin are the orthologs of SEC13 homolog (*SEC13*) and TatD DNAse domain‐containing 2 (*TATDN2*) in mammals and the coelacanth. The ghrelin of teleost fishes including *D. rerio* and the cave fish, *A. mexicanus,* is flanked downstream by *TATDN2* but upstream is flanked by a different gene annotated as *coiled coil domain‐containing 174* (*CCDC174*). While birds (*G. gallus*) possess *SEC13* upstream similar to mammals, the downstream flanking gene is interleukin‐1 receptor‐associated kinase‐like 2 (*IRAK2*).

The structural organization analyses of the ghrelin gene varied in the exon and intron counts between vertebrate lineages (Appendix S1). The analysis of protein structure revealed that the entire protein is constituted by a soluble part, and no *TMD* was predicted in the protein sequence of any analyzed species (Appendix S2). The number of *α*‐helices, *β*‐strands, and coils of the ghrelin gene in mammals, birds, amphibians, reptiles, teleosts, and coelacanth is summarized in Appendix S1. The analysis of *RSA* revealed values ranging from 2 to 8, with most of them being equal or greater than 4 (Appendix S3). There were few amino acid residues with a *RSA* value in the first *α*‐helix of the protein sequence. The main characteristic of the amino acid residues of the first helix is hydrophobicity, as compared to the other helices (consisting polar or charged residues).

### *GHS‐R* in tetrapods

Two *GHS‐Ra* isoforms were previously reported in tetrapods. One isoform results from regular pre‐mRNA splicing, while the other derives from alternative splicing of the same gene (Kaiya et al. 2013a, 2014a). As the two mammalian *GHS‐R* isoforms are encoded by the same locus, only one variant was considered in this study. A list of mammalian species possessing *GHS‐Ra* is provided in Table 1. The orthologous isoform of this receptor was found in a total number of 22 mammalian species. The synteny analyses in available genomes of human, chimpanzee, cow, pig, cat, mouse, and rat revealed that the *GHS‐Ra* isoform is flanked upstream by the tumor necrosis factor (ligand) superfamily, member 10 gene (*TNFSF10*) and downstream, by fibronectin type III domain‐containing 3B (*FNDC3BB*). The orthologs of the mammalian *GHS‐Ra* gene were located in amphibian species (*X. laevis, X. Silurana tropicalis, Hyla japonica, R. catesbeiana*) and in reptiles (*C. mydas*,* P. sinensis*,* A. sinensis*) as well as in birds (*G. gallus, M. gallopavo*) (Table 2). Similar to the mammalian *GHS‐Ra*, the flanking genes are orthologous to *FNDC3BB and TNFSF10*. The amino acid residues D99, C116, E124, M213, S217, and H280 that have been shown to play key roles in the function of *GHS‐R* are present in all tetrapod groups.

**Table 1:** *GHS‐R*a isoform in mammal vertebrates and its flanking genes

Species	Isoform	Gene ID	Protein ID	Length (aa)	Exon	Intron	Genomic location	Upstream FG	Downstream FG
*Pan troglodytes*	GHS‐Ra	ENSPTRG00000015630	ENSPTRT00000029141	366	2	1	Chrom. 3	FNDC3B‐202	TNFSF10
*Homo sapiens*	GHS‐Ra	ENSG00000121853	ENST00000241256	366	2	1	Chrom. 3	FNDC3B‐202	TNFSF10
*Gorilla gorilla*	GHS‐Ra	ENSGGOG00000024411	ENSGGOT00000026952	366	2	1	Chrom. 3	FNDC3B‐202	TNFSF10
*Nomascus leucogenys*	GHS‐Ra	ENSNLEG00000005544	ENSNLET00000007039	366	2	1	SP:GL397268.1	FNDC3B‐202	TNFSF10
*Pongo abelii*	GHS‐Ra	ENSPPYG00000014302	ENSPPYT00000016628	366	2	1	Chrom. 3	FNDC3B‐202	TNFSF10
*Macaca mulatta*	GHS‐Ra	ENSMMUG00000004194	ENSMMUT00000005956	366	2	1	Chrom. 2	FNDC3B‐202	TNFSF10
*Echinops telfairi*	GHS‐Ra	ENSETEG00000017782	ENSETET00000017782	366	3	2	GeneScaffold_2908		
*Sarcophilus harrisii*	GHS‐Ra	ENSSHAG00000008575	ENSSHAT00000009991	362	2	1	Scaffold GL849903.1	FNDC3B‐202	NCEH1
*Monodelphis domestica*	GHS‐Ra	ENSMODG00000021322	ENSMODT00000027128	332	2	1	Chrom. 7	FNDC3B‐202	TNFSF10
*Macropus eugenii*	GHS‐Ra	ENSMEUG00000002339	ENSMEUT00000002334	361	4	3	GeneScaffold_3457		
*Loxodonta africana*	GHS‐Ra	ENSLAFG00000017284	ENSLAFT00000017283	366	2	1	SuperContig scaffold_42	FNDC3B‐202	TNFSF10
*Callithrix jacchus*	GHS‐Ra	ENSCJAG00000002211	ENSCJAT00000004190	366	2	1	Chrom. 17	FNDC3B‐202	TNFSF10
*Dipodomys ordii*	GHS‐Ra	ENSDORG00000013291	ENSDORT00000013291	366	2	1	Scaffold_40905		
*Ictidomys tridecemlineatus*	GHS‐Ra	ENSSTOG00000015577	NSSTOT00000015578	366	2	1	Scaffold JH393403.1	FNDC3B‐202	TNFSF10
*Mus musculus*	GHS‐Ra	ENSMUSG00000051136	ENSMUST00000057186	364	3	2	Chrom. 3	FNDC3B‐202	TNFSF10
*Rattus norvegecus*	GHS‐Ra	ENSRNOG00000024119	ENSRNOT00000034960	364	2	1	Chrom. 2	FNDC3B‐202	TNFSF10
*Felis catus*	GHS‐Ra	ENSFCAG00000011266	ENSFCAT00000011269	366	2	1	Chrom. C2	FNDC3B‐202	TNFSF10
*Ailuropoda melanoleuca*	GHS‐Ra	ENSAMEG00000000992	ENSAMET00000001072	366	2	1	Scaffold GL192841.1	FNDC3B‐202	TNFSF10
*Mustela putorius furo*	GHS‐Ra	ENSMPUG00000017162	ENSMPUT00000017307	366	2	1	Scaffold GL896952.1	FNDC3B‐202	TNFSF10
*Myotis lucifugus* GHSR	GHS‐Ra	ENSMLUG00000000008	ENSMLUT00000000008	368	2	1	Scaffold GL429820	FNDC3B‐202	TNFSF10
*Bos taurus*	GHS‐Ra	ENSBTAG00000010874	ENSBTAT00000014446	366	2	1	Chrom.1	FNDC3B‐202	TNFSF10
*Equus caballus*	GHS‐Ra	ENSECAG00000017722	ENSECAT00000018720	366	2	1	Chrom. 19	FNDC3B‐202	TNFSF10
*Sus scrofa*	GHS‐Ra	ENSSSCG00000011754	ENSSSCT00000012865	289	1	0	Chrom. 13	TNFSF10	NCEH1
*Ochotona princeps*	GHS‐Ra	ENSOPRG00000003254	ENSOPRT00000003244	361	9	8	GeneScaffold_434	FNDC3B‐202	
*Oryctolagus cuniculus*	GHS‐Ra	ENSOCUG00000007340	ENSOCUT00000007339	366	2	1	Chrom. 14	FNDC3B‐202	TNFSF10
*Tupaia belangeri*	GHS‐Ra	ENSTBEG00000011302	ENSTBET00000011287	364	3	2	GeneScaffold_485	FNDC3B‐202	
*Cavia porcellus*	GHS‐Ra	ENSCPOG00000014041	ENSCPOT00000014183	316	6	5	Scaffold_100: 5	FNDC3B‐202	TNFSF10
*Otolemur garnettii*	GHS‐Ra	ENSOGAG00000033442	ENSOGAT00000030803	365	2	1	Scaffold GL873597.1	FNDC3B‐202	TNFSF10
*Canis familiaris*	GHS‐Ra	NSCAFG00000015376	ENSCAFT00000024389	291	2	1	Chrom. 34	FNDC3B‐202	TNFSF10

**Table 2:** *GHS‐R* isoforms in nonmammal vertebrates and their flanking genes

Species	ID or Accession	Isoform	Length (aa)	Location	Upstream FG	Downstream FG
*Aptenodytes forsteri*	XP_009286294.1	GHS‐Ra	336			
*Pygoscelis adeliae*	XP_009331878.1	GHS‐Ra	465			
*Charadrius vociferus*	XP_009882812.1	GHS‐Ra	371			
*Struthio camelus australis*	XP_009672054.1	GHS‐Ra	336			
*Acanthisitta chloris*	XP_009077327.1	GHS‐Ra	344			
*Ficedula albicollis*	XP_005051353.1	GHS‐Ra	352			
*Mesitornis unicolor*	XP_010178001.1	GHS‐Ra	352			
*Picoides pubescens*	XP_009900654.1	GHS‐Ra	352			
*Gallus gallus*	ENSGALG00000027947	GHS‐Ra	347	Chromosome 9		
*Meleagris gallopavo*	XP_010715200.1	GHS‐Ra	347			
*Chelonia mydas*	XP_007067002.1	GHS‐Ra	358			
*Chrysemys picta bellii*	XP_005299327.1	GHS‐Ra	358			
*Pelodiscus sinensis*	XP_006134697.1	GHS‐Ra	416			
*Anolis carolinensis*	ENSACAG00000016405	GHS‐Ra	355	Chromosome 3	FNDC3BB	TNFSF10
*Rana catesbeiana*	BAM11343.1	GHS‐Ra	374			
*Hyla japonica*	BAM11344.1	GHS‐Ra	371			
*Xenopus tropicalis*	ENSXETG00000023610	GHS‐Ra	359	Scaffold GL172639.1		PLD1A
*Latimeria chalumnae*	XP_006010086.1	GHS‐Ra	359			
*Clupea harengus*	XP012676703	GHS‐R1a	370			
*Lepisosteus oculatus*	XP006637866	GHS‐R1a	365			
*Carassius auratus*	BAH60672.1	GHS‐R1a	360			
*Danio rerio*	ENSDARG00000056230	GHS‐R1a	360	Chrom 2	FNDC3BB	PLD1A
*Lepisosteus oculatus*	ENSLOCT00000009083	GHS‐R1a	365	Chrom LG14	FNDC3BB	TNFSF10
*Clupea harengus*	XP007232745	GHS‐R2a	357			
*Astyanax mexicanus*	ENSAMXT00000003803	GHS‐R2a	357	Scaf KB871728.1	FNDC3BB	PLD1A
*Danio rerio*	ENSDARG00000057117	GHS‐R2a	365	Chrom 24	FNDC3BB	PLD1A
*Carassius auratus*	BAH60674.1	GHS‐R2a	367			
*Cyprinus carpio ‘jian’*	ADN05126.1	GHS‐R2a	366			
*Esox lucius*	XP_010884518.1	GHS‐R1a‐LR	391			
*Oncorhynchus mykiss*	BAF80871.1	GHS‐R1a‐LR	387			
*Gasterosteus aculeatus*	ENSGACT00000014515	GHS‐R1a‐LR	381	groupXV	FNDC3BB	PLD1A
*Takifugu rubripes*	ENSTRUT00000032893	GHS‐R1a‐LR	398	scaffold_158	FNDC3BB	PLD1A
*Tetraodon nigroviridis*	ENSTNIG00000006665	GHS‐R1a‐LR	368			
*Oreochromis niloticus*	ENSONIT00000001069	GHS‐R1a‐LR	385	Scaf. GL831153.1:	FNDC3BB	PLD1A
*Notothenia coriiceps2*	XP_010765839.1	GHS‐R1a‐LR	381			
*Xiphophorus maculatus*	ENSXMAT00000009185	GHS‐R1a‐LR	384	Scaf. JH556918.1	FNDC3BB	PLD1A
*Poecilia formosa*	ENSPFOT00000011989	GHS‐R1a‐LR	385	Scaffold KI519770	FNDC3BB	PLD1A
*Fundulus heteroclitus*	XP_012731180.1	GHS‐R1a‐LR	379			
*Oryzias latipes*	ENSORLG00000011709	GHS‐R1a‐LR	384			
*Maylandia zebra*	XP_004540224.1	GHS‐R1a‐LR	384			
*Neolamprologus brichardi*	XP_006810120.1	GHS‐R1a‐LR	384			
*Pundamilia nyererei*	XP_005736667.1	GHS‐R1a‐LR	409			
*Oreochromis niloticus*	XP_003442929.2	GHS‐R1a‐LR	409			
*Oreochromis mossambicus*	BAF80120.1	GHS‐R1a‐LR	384			
*Gadus morhua*	ENSGMOT00000014265	GHS‐R1a‐LR	377	GeneScaf_2006	FNDC3BB	PLD1A
*Cynoglossus semilaevis*	XP_008308532.1	GHS‐R1a‐LR	391			
*Acanthopagrus schlegelii*	AAN77875.1	GHS‐R1a‐LR	385			
*Dicentrarchus labrax*	DLAgn_00024700	GHS‐R1a‐LR	384	LG12	FNDC3BB	PLD1A
*Stegastes partitus*	XP_008293736.1	GHS‐R1a‐LR	483			
*Larimichthys crocea*	XP_010730499.1	GHS‐R1a‐LR	382			
*Larimichthys crocea*	XP_010739090.1	GHS‐R1a‐LR	461			
*Cynoglossus semilaevis*	XP_008316504.1	GHS‐R1a‐LR	285			
*Danio rerio*	XP_009299834.1	GHS‐Ra‐LR2a	344		PPP1R35	BAHD1
*Oreochromis niloticus*	XP_013127563.1	GHS‐Ra‐LR2a	451			
*Cynoglossus semilaevis*	XP_008329002.1	GHS‐Ra‐LR2a	530			
*Fundulus heteroclitus*	XP_012713154.1	GHS‐Ra‐LR2a	501			
*Oryzias latipes*	XP_011477016.1	GHS‐Ra‐LR2a	465		ECI1	TAC4
*Dicentrarchus labrax*	DLAgn_00195800	GHS‐Ra‐LR2a	543	LG8	ECI1	PDXDC1
*Neolamprologus brichardi*	XP_006796749.1	GHS‐Ra‐LR2a	312			
*Haplochromis burtoni*	XP_005937594.1	GHS‐Ra‐LR2a	303			
*Lepisosteus oculatus*	XP_006628149.1	GHS‐Ra‐LR2a	322		Unknown	Unknown
*Maylandia zebra*	XP_004559129.1	GHS‐Ra‐LR2a	303			
*Poecilia reticulata*	XP_008423208.1	GHS‐Ra‐LR2a	308		DIABLO	
*Takifugu rubripes*	XP_003979922.1	GHS‐Ra‐LR2a	310			
*Danio rerio*	XP_009289844.1	GHS‐Ra‐LR2b	315		DIABLO	P4HA3
*Astyanax mexicanus*	ENSAMXT00000020669	GHS‐Ra‐LR2b	338	Scaf KB871790.1	PPP1R35	CAMTA2
*Cynoglossus semilaevis*	XP_008329002.1	GHS‐Ra‐LR2b	530			
*Stegastes partitus*	XP_008278319.1	GHS‐Ra‐LR2b	384			
*Dicentrarchus labrax*	DLAgn_00035430	GHS‐Ra‐LR2b	363	LG13	FNDC3BB	RCSD1
*Oreochromis niloticus*	XP_013119981.1	GHS‐Ra‐LR2b	312		DIABLO	Uncharacterized protein
*Fundulus heteroclitus*	XP_012709453.1	GHS‐Ra‐LR2b	308			
*Pundamilia nyererei*	XP_005754504.1	GHS‐Ra‐LR2b	479			
*Oryzias latipes*	ENSORLT00000003605	GHS‐Ra‐LR2b	364	Chrom 13	FNDC3BB	ENSORLT00000003597
*Xiphophorus maculatus*	XP_005802866.1	GHS‐Ra‐LR2b	577		ECI1	TAC4
*Poecilia formosa*	XP_007573217.1	GHS‐Ra‐LR2b	576		ECI1	TAC4

### *GHS‐R1a/2a* in fishes and the coelacanth are the mammalian orthologs

Two orthologs (*GHS‐R1a* and *GHS‐R2a*) of the mammalian *GHS‐Ra* were identified in *D. rerio,* with the *GHS‐R1a* isoform comprising of 360 amino acids (aa), consisting 2 exons and 1 intron. *GHS‐R2a* is 365 aa in size with identical exon–intron arrangement. Both of these isoforms are flanked upstream by *FNDC3BB* as found for all other vertebrates but downstream by a different gene, phospholipase D1a (*PLD1A*). The *D. rerio GHS‐R1a* isoform is more similar to the orthologs of other vertebrates. *GHS‐R1a* is found in a limited number of other fish species (*Carassius auratus*,* Lepisosteus oculatus*,* Clupea harengus* and *Astyanax mexicanus*) where it is flanked by the same genes as the mammalian isoform. The *GHS‐R2a* ortholog was also found only in a limited number of teleost species such as *C. auratus* and *C. carpio* with the same flanking genes *as GHS‐R1a*.

### Identification of teleost‐specific isoform *GHS‐R1a‐LR*

Another ortholog flanked by the same genes *(FNDC3BB* and *PLD1*A) as *D. reri*o *GHS‐R1*a and *GHS‐2*a was identified in a large number of teleost species including the pufferfishes (*Takifugu rubripes* and *Tetraodon nigroviridis*), tilapias (*Oreochromis niloticus* and *Oreochromis mossambicus*), *Gasterosteu*s *aculeatus*,* Oryzias latipes*,* Gadus morhua*,* Xiphophorus maculates*,* Poecilia formosa*,* Fundulus heteroclitus*,* Dicentrarchus labrax*, and *Larimichthys crocea*. This isoform is more identical to *D. rerio GHS‐R1a* and is named as *GHS‐R1a‐Like receptor* (*GHS‐R1a‐LR*) in this study.

The order of *GHS‐R* and surrounding genes relative to each other on the forward strand of the genomic fragment is *PLD1A*‐*FNDC3BB*‐*GHSR*‐*TNFSF10* in *H. sapiens* and *L. oculatus*,* TNFSF10*‐*GHSR*‐*FNDC3BB*‐*PLD1A* in *A. carolinensis* and *G. gallus*, and *PLD1A*‐*GHSR*‐*FNDC3BB*‐(3 other genes)‐*TNFSF10* in *D. rerio*. Considering the gene direction, it is evident that the order of the genes relative to each other is identical in *H. sapiens, G. gallus, A. carolinensis, and L. oculatus*. The coordinates of the whole chromosomes in *G. gallus* and *A. carolinensis* are probably inverted, possibly at a higher level inversion. This explains the different order compared to *H. sapiens* and *L. oculatus*. In *D. rerio*, a lower level inversion of (*TNFSF10*(+), *GHS‐R*(+), *FNDC3BB*(−)) *PLD1A*(+), followed by a translocation of TNFSF10, resulted in:(*FNDC3BB*(+), *GHS‐R*(−), translocated) *PLD1A*(+). In conclusion, the difference in the downstream flanking gene between fish *GHS‐R1a*,* GHS‐R2a GHS‐R1a‐LR,* and tetrapod *GHS‐Ra* is the consequence of inversion and translocation of *TNFS10* in fishes. This arrangement appears to be common but specific to teleost species, as in the euteleost *L. oculatus*,* GHS‐R1a* is flanked downstream by *TNFS10*. Together, synteny and similarity suggest that *GHS‐R1a‐lR* is probably the same isoform as to *GHS‐R1a*, which in turn is paralogous to *GHS‐Ra*.

### Identification of teleost‐specific isoforms *GHS‐Ra‐LR2a/b*

A new isoform was named *GHS‐Ra‐like receptor 2a* (*GHS‐Ra‐LR2a*) in this study as it displays high similarity to *GHS‐R1a‐LR*. *GHS‐Ra‐LR2a* was found in a significant number of teleost species such as *L. oculatus*,* D. rerio*,* T. rubripes*,* O. latipes*,* F. heteroclitus*,* P. reticulate, O. niloticus,* and *Neolamprologus brichardi*. Table 2 summarizes the number of amino acid residues for each species and the exon and intron counts. The *GHS‐Ra‐LR2a* isoform is flanked upstream by enoyl‐CoA delta isomerase 1 (*ECI1*) and downstream by tachykinin 4 (hemokinin) gene (*TAC4*). In *D. labrax*, the downstream flanking gene is pyridoxal‐dependent decarboxylase domain‐containing protein 1 (*PDXDC1I*). The flanking genes of *GHS‐Ra‐LR2a* are also different in *D. rerio* and *A. mexicanus* where the upstream flanking gene is protein phosphatase 1 regulatory subunit 35 (*PPP1R35*) for both species, whereas the downstream gene different and are bromo adjacent homology domain‐containing 1 (*BAHD1*) and calmodulin‐binding transcription activator 2 (*CAMTA2*), respectively.

Phylogenetic analysis grouped another isoform of the fish *GHS‐R* to *GHS‐Ra‐LR2a* (Fig. 4). Thus, this gene was named *GHS‐Ra‐like receptor 2b* (*GHS‐Ra‐LR2b*) and identified in a limited number of fish species including *D. rerio*,* A. mexicanus*,* O. latipes*,* F. heteroclitus*,* P. formosa*,* X. maculate*s, and *D. labrax*. The length and exon/intron counts of *GHS‐Ra‐LR2b* are summarized in Table 2. The upstream flanking gene of this isoform is diablo, IAP‐binding mitochondrial protein (*DIABLO*) in *D. rerio*,* P. reticulate,* and *O. niloticus,* but its downstream flanking gene is prolyl 4‐hydroxylase, alpha polypeptide III (*P4HA3*), and uncharacterized proteins (Table 2). In *D. labra*x and *O. latipes*, the upstream flanking gene is *FNDC3BB* whereas the downstream flanking genes are *RCSD1* and an uncharacterized protein, respectively. *L. oculatus* isoform is flanked by two unknown genes (Table 2).

### Secondary structure characteristics of *GHS‐R* isoforms in vertebrates

Key roles in the function of *GHS‐R* are mediated by the amino acid residues D99, C116, E124, M213, S217, and H280 (Miki et al. 1992; Howard et al. 1996; Kaiya et al. 2008, 2013a, 2014a), which are present in tetrapods. While these functional amino acid residues were also found in all fish *GHS‐R* isoforms, their relative positions differed in some species either due to insertion or deletion of amino acid residues. All *GHS‐Ra* orthologs and *GHS‐R1a/2*a isoforms analyzed in this study possess seven *TMD* (Appendices 4 and 5). The *GHS‐R1a‐LR* has seven *TMDs* in all analyzed species including *G. aculeatus*,* G. morhua*,* L. crocea*,* N. brichardi*,* O. mossambicus,* and *D. labrax*, except in *X. maculates* where this isoform has exceptionally nine *TMDs*. The *GHS‐Ra‐LR2a/b* isoforms both comprised seven *TMDs* except in *O. latipes* and *L. oculatus* where *GHS‐Ra‐RL2b* has only six *TMDs*. The comparative results of the protein secondary structure, including the number of *α*‐helices, *β*‐strands, and coils predicted for each *GHS‐Ra* in each species, are indicated in Appendix S5. The number of these structural units of the *GHS‐R* protein varies between species but also between isoforms.

These different isoforms differ in the length of the second extracellular loop (*ECL2*) connecting the *TMD4* and *TMD5* which is comprised of 24‐26 amino acid (aa) residues for *GHS‐R1a/2a* and *GHS‐R*a isoforms. The *ECL2* of *GHS‐Ra‐LR1* (Appendix S4) is much larger (~36 aa residues) than that of *GHS‐R1a/2a* and *GHS‐Ra* isoforms. The *GHS‐Ra‐LR2a* and *GHS‐Ra‐LR2b* display *ECL2* lengths similar to that of *GHS‐R1a*/2a (Appendix S4) in most of the species. The *GHS‐Ra‐LR2a* is mainly characterized by a large *ICL3*, with a length ranging from 90 to up 237 aa residues. This *ICL3* is exceptionally large in *F. heteroclitus* (275 aa residues). The *GHS‐Ra‐LR2b* is characterized by an *ICL3* that is shorter (5–16 aa residues) than that of the other *GHS‐R* isoforms (Appendix S4) where it is comprised of 19–26 aa residues, respectively.

The highest *RSA* values were observed in *P. sinensis*, which also has the highest number of residues with higher *RSA* values (*RSA* ≥ 7). The lowest number of residues with *RSA* was recorded for birds (*F. albicollis* and *M. gallopavo*). *H. sapiens* and *G. gorilla* have the same number of *RSA* residues (181) and higher *RSA* values (*RSA* ≥ 7). The *GHS‐R1a* has a higher number of residues with *RSA* values (276 vs. 177 aa) compared to other fish isoforms. The number of residues with higher *RSA* values was lower in *GHS‐R1a‐LR* compared to *GHS‐Ra‐LR2a/b,* which display similar number of residues with high *RSA* values. For both ghrelin and *GHS‐R* genes, null or lower *RSA* values were observed in the *TMD*. For most of the isoforms, *RSA* were higher in the *α*‐helix and *β*‐strands structures compared to coils. The only exception was observed for *GHS‐R1*a where the higher *RSA* values were observed in coil structures.

### Phylogenetic analyses

The phylogenetic reconstructions grouped vertebrate ghrelin genes into different clades, with the main groups being supported by high bootstrap values. All teleost ghrelin genes were grouped in the same clade (Fig. 2), which is the sister group of amphibian ghrelin genes. Reptilian ghrelin genes are clustered together and form the sister group of avian ghrelin. All mammalian ghrelin genes were grouped into a same clade (Fig. 2), which is distinct from all other vertebrate lineages. The phylogenetic analysis of mammalian *GHS‐Ra* revealed minor divergence and close relation between species (Fig. 3). This is also evident from the low bootstrap values (Fig. 3). The comprehensive phylogenetic reconstruction also allowed regrouping nonmammalian *GHS‐R* into different clades which were supported by higher bootstrap values (Fig. 4). Bird and reptile *GHS‐R*a are grouped into two different clades. Amphibian and coelacanth *GHS‐Ra* are grouped into the same clade (Fig. 4). These groups are closely related to both fish *GHS‐Ra*, which are subdivided into subclades *GHS‐R1a* and *GHS‐R2a*. Another subclade (*GHS‐R1a‐LR*) that belongs to the same superclade than *GHS‐R1a* was identified in teleosts. Teleost *GHS‐R1a* and *GHS‐R1a‐LR* were grouped into the same clade, which is a sister group of *GHS‐R2a* found in a limited number of teleost species (Fig. 4). Clade *GHS‐R1a‐LR* comprised isoforms that were exclusively found in actinopterygian fishes. Among the particularities of the isoforms that constitute this clade, the *ECL2* is longer as compared to its counterpart of the group *GHS‐R2a*. Two other clades (*GHS‐Ra‐LR2a* and *GHS‐Ra‐LR2b*) are also identified in fishes from the phylogenetic reconstruction (Fig. 4). The clades of *GHS‐Ra‐LR2a* and *GHS‐Ra‐LR2b* diverged from the other fish receptors (Fig. 4). Clade *GHS‐Ra‐LR2a* is comprised of genes exclusively identified in teleost fishes, and no orthologous isoforms were found in other vertebrates. The particularity of this clade is represented by genes with *ICL3* larger than their analogous loop of the other isoforms. Clade *GHS‐Ra‐LR2b* also consists of isoforms found only in teleosts, without any orthologs in other vertebrate species. The characteristic of this clade compared to its sister group is resembled by the size of the *ICL3*, which is much shorter than in the other isoforms. The phylogenetic reconstruction including *MLN‐*R showed that fish and tetrapod *MLN‐*R are grouped in the same clade, which is a sister group of the *GHS‐Ra‐LR2*a*/GHS‐Ra‐LR2*b cluster (Appendix S6). The phylogenetic tree indicated that *MLN‐R* derived from clade B prior to *GHS‐Ra‐LR2a/b* duplication whereas tetrapod *GHS‐Ra* derived from clade A whose duplication in teleosts has resulted in *GHS‐R1a*/*GHS‐R1a‐LR*/*GHS‐R2a* (Appendix S6).

### Natural selection

**Figure 5:**
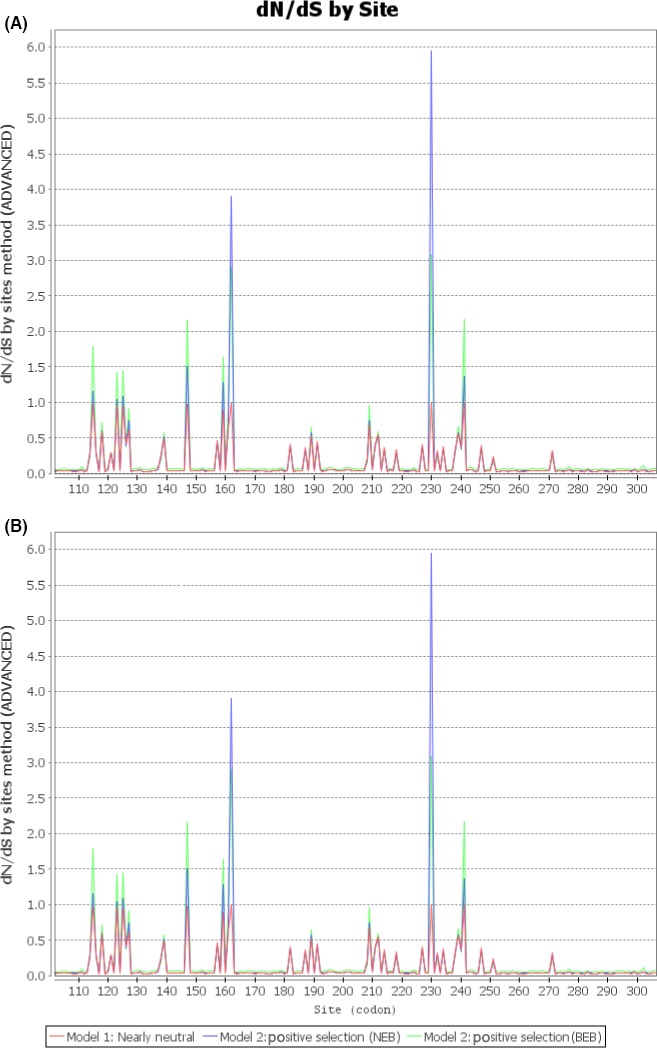
Graph sliding window *dN/dS* ratio of ghrelin genes (A) and *GHS‐R* all isoforms combined (B). The *NG* method was applied for the estimation of *dN/dS* ratios. The size of the window was set at 200 bp, with a jump of 25 bp between windows. Codons under positive selection with empirical Bayes (*NEB*) model are indicated in *blue* and in *green* with Bayes empirical Bayes (*BEB*) model. Nearly neutral codons are indicated in *red*.

The average *dN/dS* ratio, which is used to measure the selective pressure exerted on ghrelin, was significantly (*P *≤* *0.05) higher in birds and lower in amphibians compared to reptiles, mammals and fishes Table 3. Sliding window analysis applied to all ghrelin orthologs pairwise revealed regions of the protein sequences that are under positive selection. These regions are located between 110 and 250 bp (Fig. 5A), more precisely between 110 and 130 bp and around sites 146, 160, 230, and 240 bp of the nucleotide sequence. The residues under positive selection are located in regions where *α*‐helix structures are predicted, which also correspond to regions where the amino acid residues have the highest *RSA* values.

**Table 3:** Average *dN/dS* between ghrelin, *GHS‐R,* and *MBOAT* ortholog and paralogs

Cluster	Amphibians	Birds	Reptiles	Mammals	Fishes
Ghrelin	0.17 ± 0.09	0.64 ± 0.13	0.33 ± 0.04	0.29 ± 0.15	0.29 ± 0.09
*GHS‐Ra*	0.06 ± 0.03	0.10 ± 0.04	0.13 ± 0.07	0.11 ± 0.04	
*GHS‐R1a*/*2a*					0.08 ± 0.03
*GHS‐R1a‐L*					0.28 ± 0.17
*GHS‐R2a‐L*					0.29 ± 0.06
*GHS‐R‐L*					0.25 ± 0.13
GOAT (MBOAT4)	0.16 ± 0.07	0.24 ± 0.06	0.25 ± 0.04	0.24 ± 0.06	0.23 ± 0.11
MBOAT1	0.18 ± 0.05	0.18 ± 0.12	0.16 ± 0.04	0.16 ± 0.04	0.24 ± 0.06
MBOAT2	0.16 ± 0.07	0.18 ± 0.11	0.13 ± 0.06	0.10 ± 0.06	0.15 ± 0.08

The average *dN/dS* ratio of *GHS‐Ra* was lower in amphibians compared to birds, reptiles, and mammals. In teleosts, the average *dN/dS* was lower for *GHS‐R1a*/*2a* cluster compared to *GHS‐R1a‐RL*,* GHS‐R2a‐RL2a,* and *GHS‐R‐RL2b* clusters (Table 3). The average *dN/dS* ratio was significantly lower for amphibian *GHS‐Ra* and fish *GHS‐R1a/GHS‐R2a* cluster compared to mammals, birds, reptiles, and teleost *GHS‐R1a‐LR*,* GHS‐Ra‐LR2a,* and *GHS‐Ra‐LR2b* clusters. The sliding window *dN/dS* analyses of pairwise comparisons conducted along protein sequences allowed the detection of amino acid residues that are under selective constraints (Fig. 5B). The comparison of all pairwise *GHS‐R* sequences between different lineages showed similar profiles. The same regions of the proteins that are under positive selection in fishes were also found to be under positive selection in all tetrapods. Likewise, the pairwise comparisons of the sliding window between all clusters identified by the phylogenetic tree revealed similar profiles between clades, with the same codons being under positive selection at the same position of the protein sequences. The exact position of codons with *dN/dS* indicative of positive selection is highlighted in Fig. 5B. They are all located in the coding region between 110 and 250 bp, around the positions 120, 146, 160, 210, 230, and 240 codons, in regions of the proteins where the *α*‐helix and *TMD* are predicted except for those around codon 240 which, for some isoforms, is located in *ICL3*. The residues under positive selection are also located in regions of the proteins with low *RSA* values (*RSA* ≤ 1).

**Figure 6:**
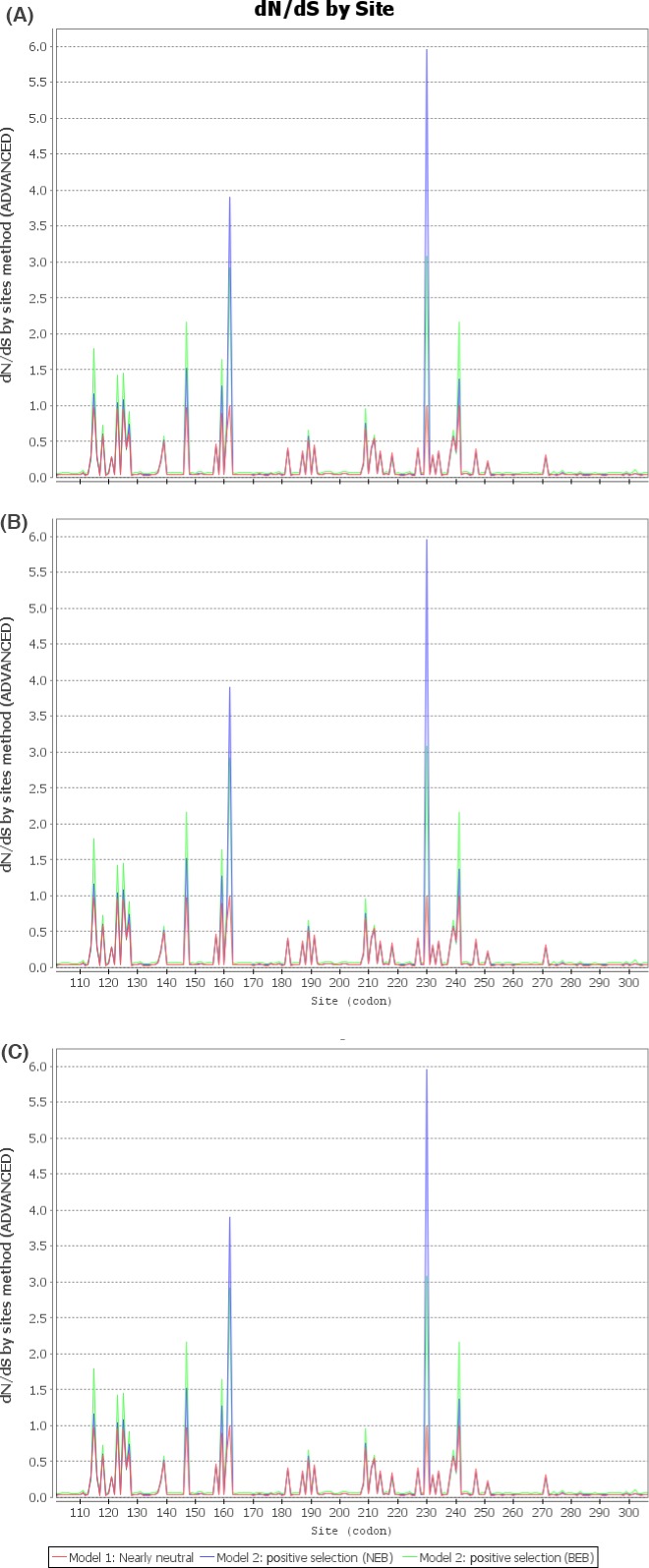
Graph *dN/* *d*S by site for (A) *MBOAT4*, (B) *MBOAT1,* and (C) *MBOAT2*.) estimated using the *NG* method. The size of the window was set at 200 bp, with a jump of 25 bp between windows. Codons under positive selection with Empirical Bayes (*NEB*) model are indicated in *blue* and in *green* with Bayes empirical Bayes (*BEB*) model. Nearly neutral codons are indicated in *red*.

The average *dN/dS* ratio of the *MBOAT4* (*GOAT*) cluster was significantly lower in amphibians compared to other lineages where it did not show differences (Table 3). Average *dN/dS* of *MBOAT4* and *MBOAT1* clusters in teleost were not significantly different. The average *dN/dS* of *MBOAT1* and *MBOAT2* were not significantly different either, except in teleost, where the average *dN/dS* of *MBOAT1* was significantly higher than that of *MBOAT2* (Table 3). The comparison with ghrelin revealed similar *dN/dS* patterns between *MBOAT4* and ghrelin in all lineages except birds. In birds, the average *dN/dS* ratio of ghrelin was higher than in the other lineages. However, bird *MBOAT4 dN/dS* is equivalent to that of reptiles, mammals, and fishes. The graph *dN/dS* by sites indicates that the same codons are under positive selection, and these codon positions are the same not only for all clusters or lineages, but also for all isoforms (Fig. 6A–C). The sites under positive selection (Fig. 6A–C) are located between codons 110–130, around codons 148, 160, 210, 230, and 240.

## Discussion

The ghrelin/ghrelin receptor hormone system is not only an important therapeutic target in translational medicine as several important physiological functions are systemically controlled in mice and men; it also represents an interesting biological system to investigate the evolutionary relationships between the unique ligand and receptor (Kaiya et al. 2011b). Uncovering the divergence and spectrum of this hormone system in the animal kingdom may assist in unraveling specific and unspecific, as well as direct and indirect functions of ghrelin. In order to shed light onto the evolution of the ghrelin/ghrelin receptor system, we found that the interaction of ghrelin and ghrelin receptor is based on a single gene in most vertebrates except teleost fishes. In the teleosts, we found several ghrelin receptor isoforms that we used to reconstruct the evolution of the ghrelin system and that we further analyzed to highlight conversed and diverse secondary structures.

It can be assumed that the novel orthologs named *GHS‐Ra‐RL2a* and *GHS‐Ra‐RL2b* in this study are also motilin receptor paralogs and/or orthologs. Interestingly, all *GHS‐R* variants found in this study were either annotated as *GHS‐R* or *GHS‐R‐like*. Moreover, the synteny analysis revealed that in most of the teleosts, the novel orthologs are flanked upstream and downstream by *ECL1* and *TAC4*, respectively. The only exceptions are *D. labrax* and *O. latipes*, where the upstream gene is *FNDC3BB*, while *RCDS1* and an unknown gene are located downstrea*m*. The vertebrate *MLN‐R,* tetrapod *GHS‐R,* and teleost *GHS‐R1a*,* GHS‐R2a,* and *GH‐R1a‐LR* have the same upstream flanking gene (*FNDC3BB*). From these synteny results, it appears that *MLN‐R* is more similar to *GHS‐Ra*,* GHS‐R1a*,* GHS‐R2a,* and *GH‐R1a‐LR* than *GHS‐Ra‐RL2a/b*. As *MLN‐R* and the ancient (previously characterized) *GHS‐R* isoforms (*GHS‐R*,* GHS‐R1a*,* GHS‐R2a,* and *GH‐R1a‐LR*) have the same upstream flanking gene in both tetrapods and teleosts, which is different from that of novel orthologs (*GHS‐Ra‐RL2/b*), it cannot be concluded from the synteny results that these novel orthologs are similar to *GHS‐R* and *MLN‐R* only in teleosts. Moreover, the synteny results indicate that *GHS‐R1a‐LR* and *GHS‐R1a* have the same upstream and downstream flanking genes (*FNDC3BB* and *PLD1A*, respectively), suggesting that *GHS‐R1a‐LR* is probably the same isoform as *GHS‐R1a*, which in turn is paralogous to tetrapod *GHS‐Ra* and teleost *GHS‐R2a*. Thus, if *GHS‐Ra‐LR1* equals *GHS‐Ra*, then *GHS‐Ra‐LR2a* and *GHS‐Ra‐LR2b* are paralogs from a fish‐specific *WGD* after their ancestral gene has derived from motilin receptor. The novel orthologs identified in this study are not derived from alternative splicing. Therefore, none of them is referred to as *GHS‐Rb*. Overall, the nomenclature given to the ancient *GHS‐R* orthologs in this study is in accordance with that of previous studies (Kaiya et al. 2013a, 2014a). Also, the nomenclature given to the novel isoforms reflects their similarity with the ancient *GHS‐R* isoforms and *MLN‐R*. Motilin receptor gene was probably duplicated as for *GHS‐R*, but the duplicates were subsequently lost. This may explain why only one *MLN‐R* isoform exists in both tetrapods and teleosts.

**Figure 7:**
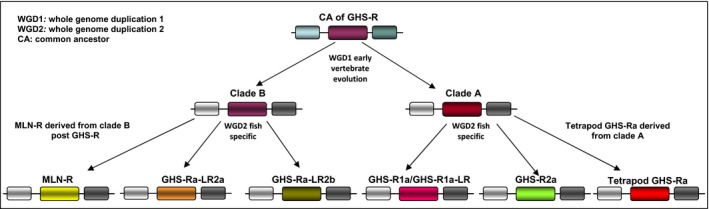
Evolutionary scenario explaining duplication events that gave rise to the different GHS‐R isoforms in vertebrates. Duplication in early vertebrate evolution (*WGD1*); fish‐specific whole‐genome duplication (*WGD2*); *GHS‐R1a‐LR* same than *GHS‐R1a*;*GHS‐R2a* lost in fish species other than Cypriniformes; (CA = Common ancestor).

The phylogenetic reconstructions of this study suggest that the ancestral gene of *GHS‐R* has undergone successive rounds of duplications that have resulted in different isoforms. While duplicated isoforms are usually lost during the course of evolution due to redundancy, the maintenance of several *GHS‐R* genes suggests some diversification of the ghrelin/ghrelin receptor system in teleosts. The evolutionary scenario that has led to the different *GHS‐R* isoforms is illustrated in Figure 7. The ancestral gene of *GHS‐R* was duplicated during early vertebrate evolution into two main clades: *Clade A* comprises *GHS‐Ra* (*GHS‐R2a/GHS‐R1a‐LR/GHS‐R1a)* orthologs in teleosts; and *Clade B* comprises *GHS‐Ra‐LR2a/GHS‐Ra‐LR2b,* both subgroups clustering in distinct clades. A teleost‐specific whole‐genome duplication of clade A has resulted to *GHS‐R1a‐LR/GHS‐R1a* and *GHS‐R2a,* with *GHS‐R1a‐LR* being the same than *GHS‐R1a. GHS‐R2a* has been subsequently lost in all teleosts except in some cypriniforme species. Thus, teleost *GHS‐R1a/GHS‐R1a‐LR* is a sister group of mammalian *GHS‐Ra* which in turn is orthologous to fish *GHS‐R2a* and tetrapod *GHS‐Ra*. The synteny results uncovered that the neighboring genes of mammal, bird, reptile, and amphibian *GHS‐Ra* are *FNDC3B* and *TNFS10*. In teleosts, *GHS‐R1a*,* GHS‐R1a‐LR,* and *GHS‐R2a* are all flanked by *FNDC3B* and *PLD1A*. However, there is a *TNFS10* gene next to *PLD1A* (at the fourth position of genes located downstream of *PLD1A*) in humans, suggesting that the *TNFS10* gene has been translocated in teleosts. This indicates that *GHS‐R1a‐LR* is the same isoform as *GHS‐R1a*, which is the paralog of *GHS‐Ra*. This interpretation of the origin of *GHS‐R1a/2a* differs from that of previous studies, which suggested that these two isoforms resulted from a duplication event that specifically occurred in some cypriniforme species (Kaiya et al. 2013a, 2014a). We suggest that there is confusion on the origin of *GHS‐Ra2* from a recent *WGD* (tetraploidization) in fishes. From completely sequenced genomes, *GHS‐R2a* is only present in carp (*Cyprinus*) but not in grass carp, suggesting that *GHS‐R2a* is highly specific to the genus *Cyprinus* and possibly some other cypriniformes. While the similarity search indicated that *GHS‐R1a* and *GHS‐R1a‐LR* are more similar compared to other isoforms, the structural protein results showed that the *ECL2* length differs between these two isoforms. This may be seen as a contradiction of the above interpretation (*GHS‐R1a* same variant as *GHS‐R1a‐LR*), but the larger *ECL2* of *GHS‐R1a‐LR* may have resulted from structural changes that specifically have affected this isoform (Kaiya et al. 2013a). The presence of only one *GHS‐Ra* isoform in the elephant shark, *Callorhinchus milii,* genome supports the idea that the duplication specifically occurred in teleost fishes. The *GHS‐R1a* and *GHS‐R2a* isoforms were identified on corresponding chromosomes 2 and 24 in zebrafish, which provides evidence that they originated from *WGD* (Meyer and Schartl 1999; Jaillon et al. 2004; Kaiya et al. 2013a, 2014a). This interpretation is also in agreement with the synteny between homologous genomic regions harboring the two isoforms from this duplication event, which are still conserved. The same *WGD* has affected clade B and has resulted in *GHS‐Ra‐LR2a* and *GHS‐Ra‐LR2b*. We believe that the same *WGD* that has resulted to *GHS‐R1a/GHS‐R1a‐LR* and *GHS‐R2a* has also resulted to *GHS‐Ra‐LR2a* and *GHS‐Ra‐LR2b*, which constitute the clade B. Although the *GHS‐Ra‐LR2a* and *GHS‐Ra‐LR2b* isoforms still remain in the genome, the micro‐synteny of genomic regions harboring them is no longer conserved, probably because these regions have undergone genomic re‐arrangements including insertions, deletions, and translocations that may have altered sequence homology signals. This may explain why *GHS‐Ra‐LR2a* and *GHS‐Ra‐LR2b* isoforms were not detected in corresponding genomic regions in any of the fish species. *GHS‐R1a*,* GHS‐R2a,* and *GHS‐Ra‐LR2a* have an *ECL2* with similar length, which is also equivalent to the length of *MLN‐R ECL2*, suggesting that *ECL2* length is a characteristics that may have been inherited from the common ancestral gene of *GHS‐R* and *MLN‐R*. The large *ECL2* length of *GHS‐R1a‐LR* has probably resulted from structural changes that have specifically affected these two *GHS‐R* isoforms after duplication. Similarly, the exceptionally larger *ICL3* of *GHS‐Ra‐LR2a* was probably acquired through structural changes that have specifically occurred in this *GHS‐R* isoform after duplication. By contrast, the shorter *ICE3* of *GHS‐Ra‐LR2b* seems to have common characteristics with *MLN‐R*, which also have one *ICE3* with a similar length. If *MLN‐R* and *GHS‐R* have both been considered as belonging to the motilin–ghrelin protein gene family, it has never been demonstrated that *MLN‐R* shares more similarity with *GHS‐Ra‐LR2a/b* isoforms that were not clearly characterized. The phylogenetic reconstruction showed that fish *MLN‐R* and *GHS‐R1a‐LR2*/*GHS‐Ra‐LR* are sister groups and certainly resulted from duplication of the same common ancestral gene.

The biological activities of ghrelin are mediated by *GHS‐R*. However, ghrelin acylation by ghrelin O‐acyltransferase (*GOAT*) enzyme is required for the accomplishment of all its physiological activities. The ghrelin amino acid residues Gly‐Ser‐Ser(*n*‐octanoyl)‐Phe‐NH2 are required for the activation of ghrelin (Kojima and Kangawa 2005) and are conserved among most species analyzed in this study, except in few species including the amphibian *R. catesbeiana,* where Ser3 was replaced by Thr3. While the conservation of these essential amino acid residues in all lineages is indicative of common functions in vertebrates, the replacement of Ser by Thr may have altered the properties of ghrelin gene as experimental replacement of Ser3 by Leu led to a complete inhibition of ghrelin gene activity (Matsumoto et al. 2001; Delporte 2013). Ser3 > Thr is not amphibian‐specific, and *R. catesbeiana* may be an exception.

The functionality of *GHS‐R* isoforms is not experimentally confirmed here, but the structural properties shared by all variants suggest that they may all be responsive to the ghrelin gene. The key residue D99 is located in the *TMD2* of *GHS‐R*a, *GHS‐R1*a, *GHS‐R2*a, and *GHS‐R*a‐*LR2b* isoforms whereas C116 and E124 on one hand and M213 and S217 on the other hand are located in *TMD3* and *TMD5* of the same isoforms, respectively. The H280 residue is located in *TMD6* of the above‐mentioned isoforms, and in *TMD7* of the *GHS‐Ra‐LR2a* isoform. The location of these key residues on the same structural components, despite slight differences in positions, suggests that they have not altered the properties of *GHS‐R*. Taken together, these results indicate that all *GHS‐R* isoforms have preserved the basic functions of the ancestral ghrelin receptor gene.

Given the conservation of amino acids required for ghrelin acylation, all *GHS‐R* isoforms possess key residues that play crucial roles in receptor binding, strongly supporting the coevolution of the ligand/receptor pair. Despite sequence divergence, the functional key residues are preserved by all isoforms and positional shades, which probably have resulted from inversions or deletions, particularly in the *GHS‐R* isoforms. Ghrelin, *GOAT,* and *GHS‐R* are physiologically and biochemically interlinked (Gutierrez et al. 2008; Yang et al. 2008a, 2008b). There is a functional triangle between these three genes that might be reflected in their evolutionary profiles (Yang et al. 2008b). Coevolution between genes can result either from their proximity in the genome or reflect functional relationships (Tillier and Charlebois 2009b; Chan et al. 2013). Neighboring genes can coevolve due to the action exerted by local evolutionary factors, such as the existence of genomic regions with differential recombination activities and the density of chromatin in the regions where these genes are located (Chan et al. 2013). It has been demonstrated that evolutionary events such as retrotransposition are regulated by the chromatin status, which influences the mediating L1 element activity (Seleme et al. 2006). The identification of coevolution signals between distant loci could reflect an ancestral genomic rearrangement that may have resulted in initial neighboring genes being distantly located in the genome after rearrangements (Zhang and Gaut 2003; Koszul et al. 2004). Coevolution interactions also include gene gain or loss that can be inferred from phylogenetic reconstruction profiles and conserved synteny analyses (Chan et al. 2013; Borges et al. 2015). Our synteny results did not show signals of ancestral genomic rearrangements that may have altered the proximity of *GHS‐R*. Likewise, it is unlikely that *GHS‐R* isoforms were initially proximately located in the genome because they did not originate from tandem duplications that result in paralogs being adjacent in genome. The comparisons of substitution ratios (all isoforms combined) showed similar profiles of sliding window *dN/dS* between lineages, with the same codon positions being under positive selection. Similar results were observed from *GHS‐R* clade comparisons of sliding window *dN/dS*, which showed same the profiles between clusters, which is also similar to the patterns observed between lineages. These codons under positive selection would not affect the receptor functionality or promote functional differences because they all fall within *TMDs* and are neither lineage‐ nor isoform‐specific. They are found in all *GHS‐R* clusters, which argues against the existence of specific functional constraints that may have altered the original function or led to novel functions exclusive to any of these isoforms. Although recent studies have demonstrated the existence of correlation between *RSA* and the evolutionary rate of proteins (Shaytan et al. 2009; Tien et al. 2013), we did not identify a linear relationship between *RSA* and *dN/dS* ratio. The comparison of *dN/dS* patterns between *MBOAT* isoforms and ghrelin indicated that *MBOAT4* selection patterns are more similar to ghrelin selection profiles. Although not strong, there is signal of coevolution between ghrelin and *MBOAT4* compared to *MBOAT1/2*. However, the graph of *dN/dS* by sites shows the same amino acid residues under positive selection in all *MBOAT* isoforms. Therefore, the comparison of the selection patterns does not allow to draw definitive conclusions concerning why ghrelin specifically binds *MBOAT4*, but not *MBOAT1/2* isoforms. This suggests that there must exist other factors allowing *MBOAT4*/ghrelin specificity.

## Conclusion

The combination of similarity‐based BLAST search, phylogenetic reconstructions, conserved synteny, and protein structure analyses appeared to be the best approach for an exhaustive clarification of the evolutionary history of gene families. In addition to confirming the presence of a single ghrelin locus in vertebrates, one *GHS‐R* isoform in all tetrapods and three isoforms previously characterized in fishes, we identified two new *GHS‐R* variants in teleosts. The combined conserved synteny analyses and phylogenetic reconstructions showed that two of fish *GHS‐Rs*, namely *GHS‐R1a* and *GHS‐R1a‐LR* previously described as different isoforms, are the same variant. *GHS‐R1a* and *GHS‐R1a‐LR* differed only from the larger *ECL2* length of *GHS‐R1a‐LR* that has probably resulted from structural changes that have specifically occurred in species having this variant. The phylogenetic reconstruction, together with conserved synteny analyses, showed that all *GHS‐R* isoforms have resulted from different *WGD*, some of which were specific to teleosts. The retention of *GHS‐R* isoforms in teleosts after duplications and their functional diversification offer excellent opportunities for investigating neo‐ and/or subfunctionalization following gene duplications, processes that are responsible for the functional divergence and diversification of protein gene families. The most commonly characterized *GHS‐R* isoforms (*GHS‐Ra*,* GHS‐R1a,* and *GHS‐R2a*) share common functions with *MLN‐R*. From the phylogenetic reconstructions, it can be expected that *MLN‐R* shares more functionalities with *GHS‐Ra‐RL2a* and *GHS‐Ra‐LR2b* isoforms, to which it is more closely related. The identification of new *GHS‐R* isoforms and the clarification of the evolution history of this receptor group provide further insights for studies on structure–function relationships and may assist in determining the physiological role of the ghrelin/*GHS‐R* system.

## Conflict of Interest

None declared.

## Supporting information


**Appendix S1.** Ghrelin genes in vertebrates: Accession number, exon/intron structure, and secondary sericulture

**Appendix S2.** Secondary structure of ghrelin gene in vertebrate lineages

**Appendix S3**. Relative solvent accessibility (*RSA*) of secondary ghrelin gene in vertebrate lineages.

**Appendix S4.** Secondary structure of *GHS‐R* isoforms.

**Appendix S5**. Composition of *GHS‐R* secondary structure

**Appendix S6.** GHS‐R phylogenetic tree including MLN‐R.
