# Supplementary material for: Diversification and coevolution of the ghrelin/growth hormone secretagogue receptor system in vertebrates
Source: Ecol Evol. 2016 Mar 14;6(8):2516–35. doi: 10.1002/ece3.2057 (PMC4797157; doi:10.1002/ece3.2057)
Supplement: Supplementary file 1 — Appendix S1. Ghrelin genes in vertebrates: Accession number, exon/intron structure, and secondary sericulture [file ECE3-6-2516-s001.doc]

| **Species** | **ID or Accession** | **Exon** | **Intron** |  | **α-helix** | **β-strands** | **coils** |
| --- | --- | --- | --- | --- | --- | --- | --- |
| ***Mammalians*** |  |  |  |  |  |  |  |
| Cercocebus atys | XP_011915522.1 |  |  |  |  |  |  |
| Mandrillus leucophaeus | XP_011849522.1 |  |  |  |  |  |  |
| Colobus palliatus | XP_011807674.1 |  |  |  |  |  |  |
| Pan troglodytes | XP_003339080.1 | 6 | 5 |  |  |  |  |
| Ailuropoda melanoleuca | ABZ05755.1 |  |  |  |  |  |  |
| Mesocricetus auratus | XP_005066190.2 |  |  |  |  |  |  |
| Cricetulus griseus | XP_007652052.1 |  |  |  |  |  |  |
| Rattus rattus | EDL91557.1 | 4 | 3 |  |  |  |  |
| Mus musculus | EDK99497.1 | 5 | 4 |  | 4 | 2 | 5 to 6 |
| Canis lupus familiaris | BAC75929.1 |  |  |  |  |  |  |
| Sus scrofa | BAL41787.1 |  |  |  |  |  |  |
| Bos taurus | AAX89508.1 | 4 | 3 |  |  |  |  |
| Bubalus bubalis | AGC92267.1 |  |  |  |  |  |  |
| Ovis aries | NP_001009721.1 |  |  |  |  |  |  |
| Capra hircus | BAD34669.1 |  |  |  |  |  |  |
| Sarcophilus harrisii | XP_012399062.1 |  |  |  |  |  |  |
| Homo sapiens | ADM33790.1 |  |  |  |  |  |  |
| ***Amphibians/Coelacanth*** |  |  |  |  |  |  |  |
| Xenopus laevis | NP_001267573.1 | 3 | 2 |  |  |  |  |
| Xenopus Silurana tropicalis | XP_004914185.1 | 4 | 3 |  |  |  |  |
| Latimeria chalumnae | XP_005997884.1 |  |  |  | 3 | 2 | 7 |
| Rana catesbeiana | Q90W22.1 |  |  |  | 3 to 4 | 2 | 5 to 7 |
| Hyla japonica | BAM29301.1 |  |  |  |  |  |  |
| ***Fishes*** |  |  |  |  |  |  |  |
| Danio rerio | NP_001077341.1 | 5 | 4 |  |  |  |  |
| Clupea harengus | XP_012680274.1 | 4 | 3 |  |  |  |  |
| Astyanax mexicanus | XP_007254336.1 | 4 | 3 |  |  |  |  |
| Oncorhynchus mykiss | NP_001118060.1 |  |  |  |  |  |  |
| Pundamilia nyererei | XP_005741400.1 |  |  |  |  |  |  |
| Maylandia zebra | XP_004544940.1 | 4 | 3 |  |  |  |  |
| Oreochromis niloticus | XP_003441511.1 | 4 | 3 |  | 2 | 2 | 5 |
| Neolamprologus brichardi | XP_006807654.1 |  |  |  |  |  |  |
| Stegastes partitus | XP_008292605.1 |  |  |  |  |  |  |
| Gasterosteus aculeatus | ENSGACP00000000983 | 3 | 2 |  |  |  |  |
| Larimichthys crocea | NP_001290260.1 |  |  |  |  |  |  |
| Notothenia coriiceps | XP_010778429.1 |  |  |  |  |  |  |
| Dicentrarchus labrax | DLAgn_00092840 |  |  |  |  |  |  |
| Lepisosteus oculatus | XP_006630780.1 | 3 | 2 |  | 3 | 2 | 6 |
| ***Reptiles*** |  |  |  |  |  |  |  |
| Chelonia mydas | XP_007060001.1 | 5 | 4 |  |  |  |  |
| Pelodiscus sinensis | XP_006132884.1 | 4 | 3 |  | 2 | 2 | 5 to 6 |
| Alligator sinensis | XP_006024197.1 | 4 | 3 |  |  |  |  |
| ***Avians*** |  |  |  |  |  |  |  |
| Columba livia | NP_001269742.1 |  |  |  |  |  |  |
| Gallus gallus | NP_001001131.1 | 5 | 4 |  |  |  |  |
| Meleagris gallopavo | XP_003210257.1 | 5 | 4 |  |  |  |  |
| Anser cygnoides domesticus | XP_013037811.1 | 3 | 2 |  |  |  |  |
| Leptosomus discolor | XP_009946397.1 |  |  |  | 2 | 2 to 3 | 6 |
| Balearica regulorum gibbericeps | XP_010300087.1 |  |  |  |  |  |  |
| Nipponia nippon | XP_009468199.1 |  |  |  |  |  |  |
| Charadrius vociferus | XP_009881169.1 |  |  |  |  |  |  |
| Fulmarus glacialis | XP_009577355.1 |  |  |  |  |  |  |
