# Supplementary figures and images for: Diversification and coevolution of the ghrelin/growth hormone secretagogue receptor system in vertebrates
Source: Ecol Evol. 2016 Mar 14;6(8):2516–35. doi: 10.1002/ece3.2057 (PMC4797157; doi:10.1002/ece3.2057)

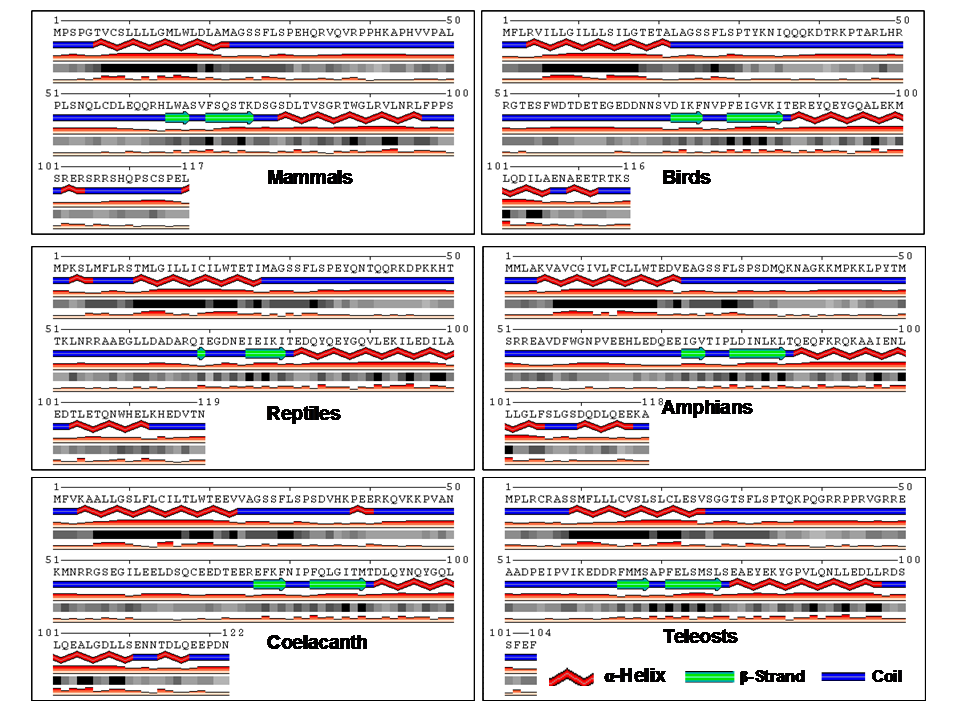

Supplement: Supplementary file 2 — Appendix S2. Secondary structure of ghrelin gene in vertebrate lineages [file ECE3-6-2516-s002.tiff]

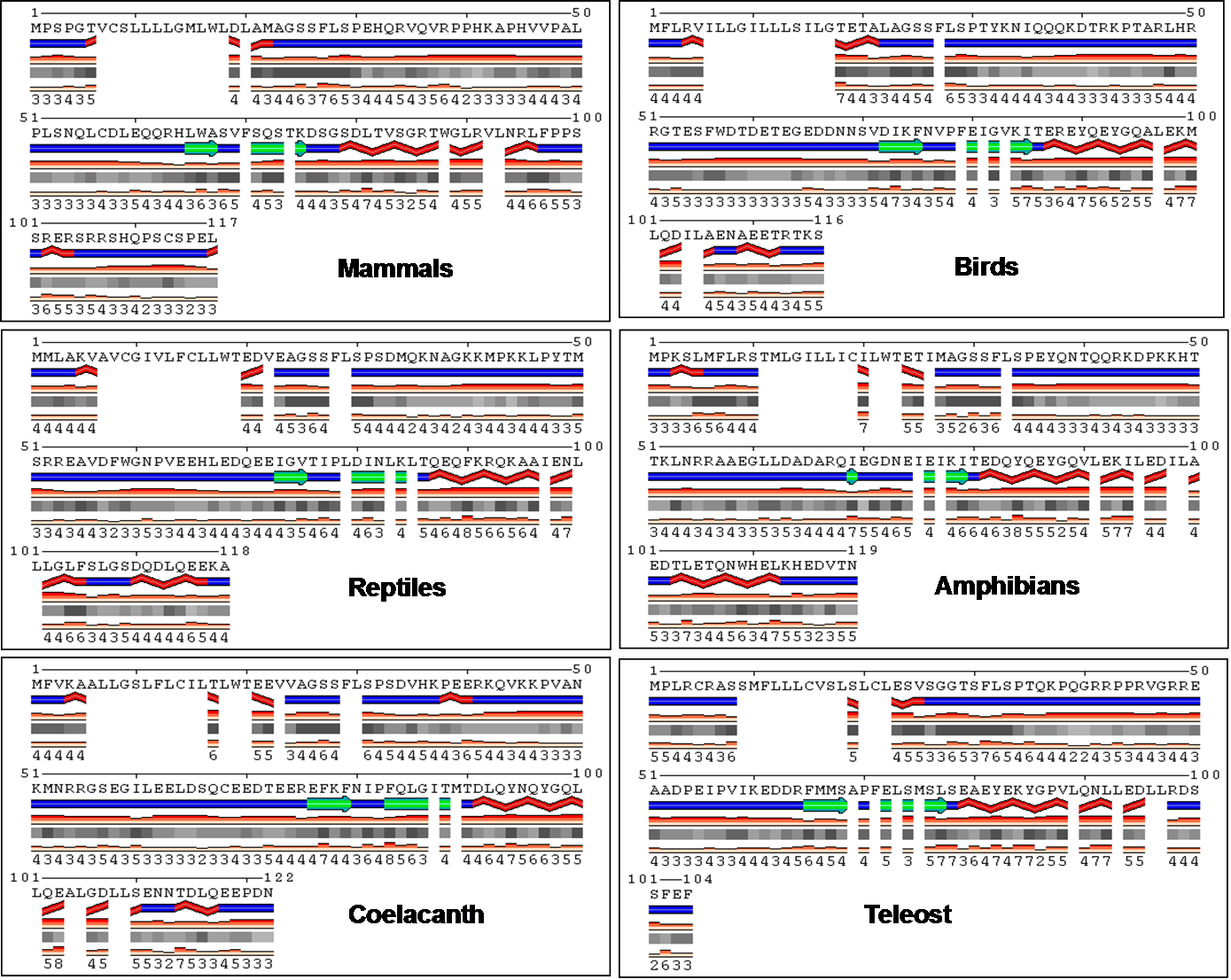

Supplement: Supplementary file 3 — Appendix S3. Relative solvent accessibility (RSA) of secondary ghrelin gene in vertebrate lineages. [file ECE3-6-2516-s003.tiff]

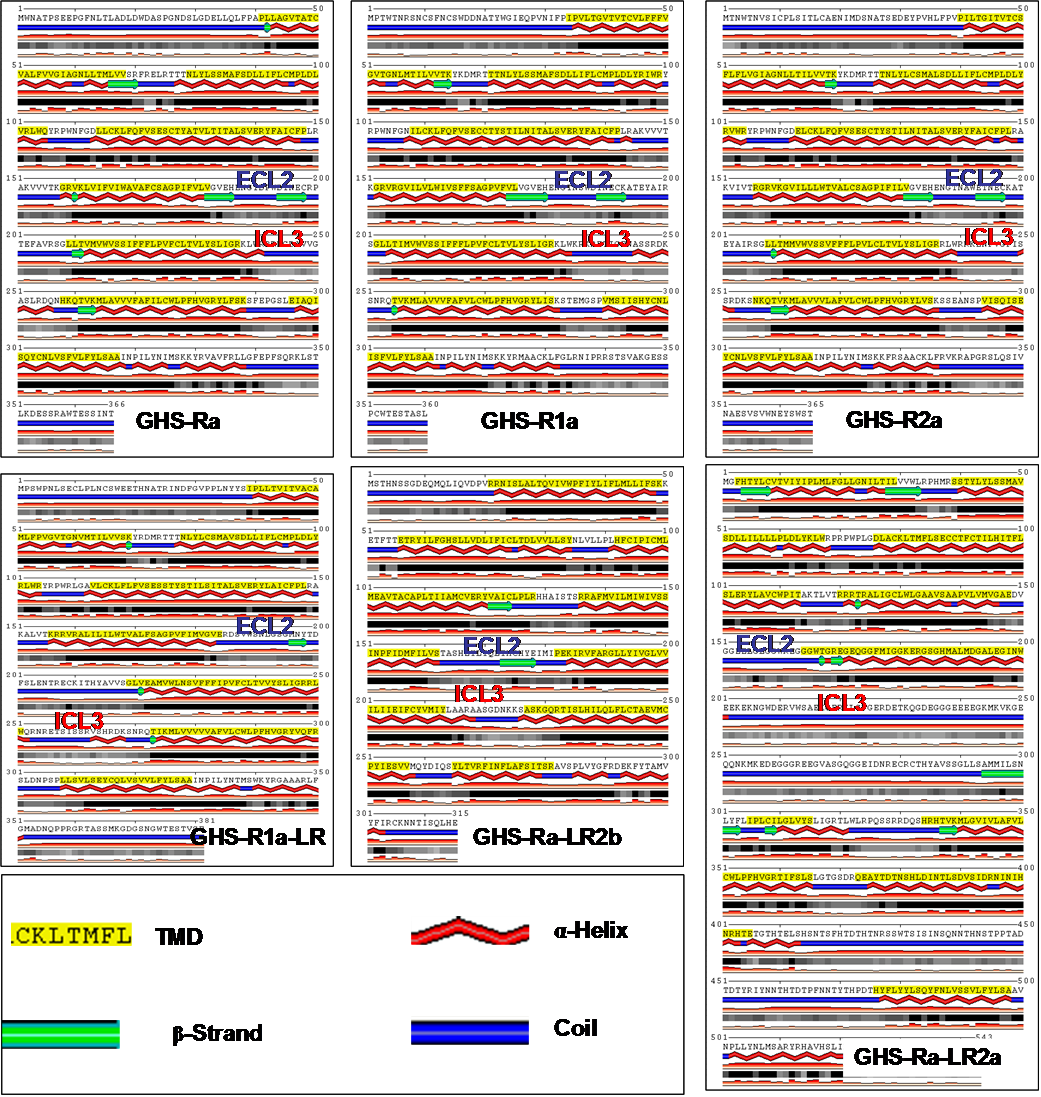

Supplement: Supplementary file 4 — Appendix S4. Secondary structure of GHS‐R isoforms. [file ECE3-6-2516-s004.tiff]
