# Supplementary material for: Diversification and coevolution of the ghrelin/growth hormone secretagogue receptor system in vertebrates
Source: Ecol Evol. 2016 Mar 14;6(8):2516–35. doi: 10.1002/ece3.2057 (PMC4797157; doi:10.1002/ece3.2057)
Supplement: Supplementary file 5 — Appendix S5. Composition of GHS‐R secondary structure [file ECE3-6-2516-s005.doc]

| ***Species*** | ***GHS-R isoform*** |  | ***Protein***  ***structure*** |  |  |  | ***RSA*** |  |
| --- | --- | --- | --- | --- | --- | --- | --- | --- |
|  |  | ***TMD*** | **α−helix** | ***β−strand*** | ***Coil*** | ***≥ 3≤ 6*** | ***≥ 7≤ 9*** | ***Total*** |
| *Latimeria chalumnae* | *GHS-Ra* | 7 | 13 | 4 | 15 | 162 | 15 | 177 |
| *Chrysemys picta bellii* | *GHS-Ra* | 7 | 11 | 4 | 13 | 154 | 13 | 167 |
| *Ficedula_albicollis* | *GHS-Ra* | 7 | 12 | 4 | 13 | 147 | 15 | 162 |
| *Meleagris gallopavo* | *GHS-Ra* | 7 | 12 | 5 | 14 | 144 | 16 | 160 |
| *Pelodiscus sinensis* | *GHS-Ra* | 7 | 14 | 6 | 17 | 211 | 18 | 229 |
| *Xenopus tropicalis* | *GHS-Ra* | 7 | 11 | 4 | 13 | 154 | 17 | 171 |
| *Hyla japonica* | *GHS-Ra* | 7 | 11 | 7 | 13 | 168 | 14 | 182 |
| *Mus musculus* | *GHS-Ra* | 7 | 11 | 4 | 13 | 171 | 15 | 186 |
| *Rattus norvegecus* | *GHS-Ra* | 7 | 12 | 4 | 14 | 142 | 14 | 156 |
| *Gorilla gorilla* | *GHS-Ra* | 7 | 13 | 7 | 14 | 164 | 17 | 181 |
| *Homo sapiens* | *GHS-Ra* | 7 | 13 | 7 | 14 | 164 | 17 | 181 |
| *Dario rerio* | *GHS-R1a* | 7 | 13 | 4 | 15 | 156 | 120 | 276 |
| *Lepisosteus oculatus* | *GHS-R1a* | 6 | 11 | 4 | 13 | 112 | 13 | 125 |
| *Danio rerio* | *GHS-R2a* | 7 | 13 | 5 | 15 | 162 | 15 | 177 |
| *Carassius auratus* | *GHS-R2a* | 7 | 14 | 5 | 15 | 188 | 15 | 203 |
| *Xiphophorus maculates* | *GHS-R1a-LR* | 9 | 17 | 5 | 18 | 337 | 19 | 356 |
| *Gasterosteus aculeatus* | *GHS-R1a-LR* | 7 | 11 | 4 | 13 | 175 | 13 | 188 |
| *Gadus morhua* | *GHS-R1a-LR* | 7 | 11 | 3 | 13 | 169 | 12 | 181 |
| *Larimichthys crocea* | *GHS-R1a-LR* | 7 | 10 | 4 | 12 | 178 | 15 | 193 |
| *Neolamprologus brichardi* | *GHS-R1a-LR* | 7 | 12 | 3 | 11 | 181 | 13 | 194 |
| *Oreochromis mossambicus* | *GHS-R1a-LR* | 7 | 12 | 2 | 13 | 179 | 13 | 192 |
| *Dicentrarchus labrax* | *GHS-R1a-LR* | 7 | 11 | 4 | 13 | 178 | 12 | 190 |
| *Danio rerio* | *GHS-Ra-LR2a* | 7 | 10 | 2 | 12 | 116 | 18 | 134 |
| *Takifugu rubripes* | *GHS-Ra-LR2a* | 7 | 10 | 2 | 12 | 115 | 14 | 129 |
| *Oryzias latipes* | *GHS-Ra-LR2a* | 6 | 12 | 2 | 16 | 107 | 74 | 181 |
| *Neolamprologus brichardi* | *GHS-Ra-LR2a* | 7 | 12 | 2 | 15 | 113 | 14 | 127 |
| *Oreochromis niloticus* | *GHS-Ra-LR2a* | 7 | 9 | 4 | 12 | 112 | 13 | 125 |
| *Fundulus heteroclitus* | *GHS-Ra-LR2a* | 7 | 12 | 2 | 15 | 95 | 18 | 113 |
| *Dicentrarchus labrax* | *GHS-Ra-LR2a* | 7 | 12 | 4 | 14 | 165 | 16 | 181 |
| *Danio rerio* | *GHS-Ra-LR2b* | 7 | 11 | 4 | 15 | 141 | 19 | 160 |
| *Astyanax mexicanus* | *GHS-Ra-LR2b* | 6 | 12 | 10 | 15 | 140 | 18 | 158 |
| *Oreochromis niloticus* | *GHS-Ra-LR2b* | 6 | 12 | 10 | 15 | 305 | 15 | 320 |
| *Oryzias latipes* | *GHS-Ra-LR2b* | 7 | 13 | 7 | 17 | 264 | 15 | 279 |
| *Dicentrarchus labrax* | *GHS-Ra-LR2b* | 7 | 12 | 2 | 14 | 260 | 17 | 277 |
| *Fundulus heteroclitus* | *GHS-Ra-LR2b* | 7 | 15 | 5 | 18 | 324 | 22 | 346 |
|  |  |  |  |  |  |  |  |  |
