# Supplementary material for: Diversification and coevolution of the ghrelin/growth hormone secretagogue receptor system in vertebrates
Source: Ecol Evol. 2016 Mar 14;6(8):2516–35. doi: 10.1002/ece3.2057 (PMC4797157; doi:10.1002/ece3.2057)
Supplement: Supplementary file 6 — Appendix S6. GHS‐R phylogenetic tree including MLN‐R. [file ECE3-6-2516-s006.pdf]

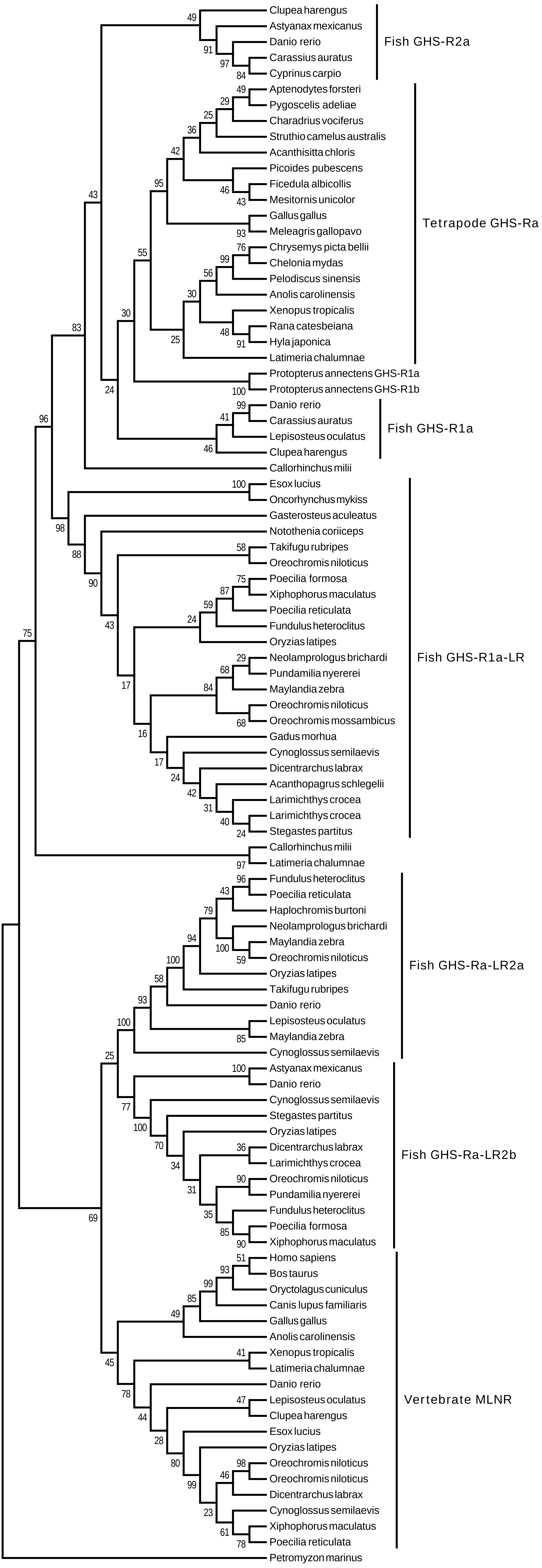

Clupea harengus
Astyanax mexicanus
Danio rerio
Carassius auratus
Cyprinus carpio
Fish GHS-R2a
Aptenodytes forsteri
Pygoscelis adeliae
Charadrius vociferus
Struthio camelus australis
Acanthisitta chloris
Picoides pubescens
Ficedula albicollis
Mesitornis unicolor
Gallus gallus
Meleagris gallopavo
Chrysemys picta bellii
Chelonia mydas
Pelodiscus sinensis
Anolis carolinensis
Xenopus tropicalis
Rana catesbeiana
Hyla japonica
Latimeria chalumnae
Tetrapode GHS-Ra
Protopterus annectens GHS-R1a
Protopterus annectens GHS-R1b
Danio rerio
Carassius auratus
Lepisosteus oculatus
Clupea harengus
Fish GHS-R1a
Callorhinchus milii
Esox lucius
Oncorhynchus mykiss
Gasterosteus aculeatus
Notothenia coriiceps
Takifugu rubripes
Oreochromis niloticus
Poecilia formosa
Xiphophorus maculatus
Poecilia reticulata
Fundulus heteroclitus
Oryzias latipes
Neolamprologus brichardi
Pundamilia nyererei
Maylandia zebra
Oreochromis niloticus
Oreochromis mossambicus
Gadus morhua
Cynoglossus semilaevis
Dicentrarchus labrax
Acanthopagrus schlegelii
Larimichthys crocea
Larimichthys crocea
Stegastes partitus
Fish GHS-R1a-LR
Callorhinchus milii
Latimeria chalumnae
Fundulus heteroclitus
Poecilia reticulata
Haplochromis burtoni
Neolamprologus brichardi
Maylandia zebra
Oreochromis niloticus
Oryzias latipes
Takifugu rubripes
Danio rerio
Lepisosteus oculatus
Maylandia zebra
Cynoglossus semilaevis
Fish GHS-Ra-LR2a
Astyanax mexicanus
Danio rerio
Cynoglossus semilaevis
Stegastes partitus
Oryzias latipes
Dicentrarchus labrax
Larimichthys crocea
Oreochromis niloticus
Pundamilia nyererei
Fundulus heteroclitus
Poecilia formosa
Xiphophorus maculatus
Fish GHS-Ra-LR2b
Homo sapiens
Bos taurus
Oryctolagus cuniculus
Canis lupus familiaris
Gallus gallus
Anolis carolinensis
Xenopus tropicalis
Latimeria chalumnae
Danio rerio
Lepisosteus oculatus
Clupea harengus
Esox lucius
Oryzias latipes
Oreochromis niloticus
Oreochromis niloticus
Dicentrarchus labrax
Cynoglossus semilaevis
Xiphophorus maculatus
Poecilia reticulata
Vertebrate MLNR
Petromyzon marinus
